# Genetic and demographic vulnerability of adder populations: Results of a genetic study in mainland Britain

**DOI:** 10.1371/journal.pone.0231809

**Published:** 2020-04-20

**Authors:** Sarah Ball, Nigel Hand, Faye Willman, Christopher Durrant, Tobias Uller, Katja Claus, Joachim Mergeay, Dirk Bauwens, Trenton W. J. Garner

**Affiliations:** 1 Institute of Zoology, Zoological Society of London (ZSL), London, England, United Kingdom; 2 Central Ecology, Ledbury, Herefordshire, England, United Kingdom; 3 Royal Veterinary College, London, England, United Kingdom; 4 Department of Zoology, University of Oxford, Oxford, England, United Kingdom; 5 Department of Biology, Lund University, Lund, Sweden; 6 Department of Biology, University of Antwerp, Wilrijk, Belgium; 7 Research Institute for Nature and Forest (INBO), Geraardsbergen, Belgium; Leibniz-Institute of Freshwater Ecology and Inland Fisheries, GERMANY

## Abstract

Genetic factors are often overlooked in conservation planning, despite their importance in small isolated populations. We used mitochondrial and microsatellite markers to investigate population genetics of the adder (*Vipera berus)* in southern Britain, where numbers are declining. We found no evidence for loss of heterozygosity in any of the populations studied. Genetic diversity was comparable across sites, in line with published levels for mainland Europe. However, further analysis revealed a striking level of relatedness. Genetic networks constructed from inferred first degree relationships suggested a high proportion of individuals to be related at a level equivalent to that of half-siblings, with rare inferred full-sib dyads. These patterns of relatedness can be attributed to the high philopatry and low vagility of adders, which creates high local relatedness, in combination with the polyandrous breeding system in the adder, which may offset the risk of inbreeding in closed populations. We suggest that reliance on standard genetic indicators of inbreeding and diversity may underestimate demographic and genetic factors that make adder populations vulnerable to extirpation. We stress the importance of an integrated genetic and demographic approach in the conservation of adders, and other taxa of similar ecology.

## Introduction

Population genetics remain overlooked in conservation planning [1], although genetic factors may lead to population extinction even after other threats have been addressed [2]. Loss of genetic diversity and inbreeding depression represent the primary genetic threats [3], with the potential to contribute to an extinction vortex [4].

Tingley et al [5] have stressed the importance of addressing the optimal genetic management of small isolated reptile populations. The adder *Vipera berus* (Linnaeus, 1758) is a terrestrial snake with an extremely wide geographic range [6], which accounts for its IUCN Red List status as “Least Concern”, although with a decreasing population trend [7, 8]. Adders, like many other temperate snakes, are viviparous with low fecundity, low vagility and high philopatry [9, 10], life-history traits that render them vulnerable to local extinction [11, 12]. The negative outlook for adder populations is exacerbated by snakes being among the least popular terrestrial vertebrates, more likely to be targets of intentional killing than of conservation management [13]. The potential risk of inbreeding depression in adders is highlighted by an isolated adder population in Sweden, in which a decline in numbers was associated with stillbirths and deformities, and a reduction in genetic diversity, all of which responded to the introduction of adult males from a large outbred population [14–16]. Újvári et al [17] have similarly reported low juvenile survival and birth deformities with reduced genetic diversity and increased homozygosity in fragmented populations of the congeneric Hungarian meadow viper, *V*. *ursinii rakosiensis*.

Small population size is an important factor in loss of genetic diversity, exacerbated by bottleneck events. This has led to the concept of a minimum viable population size [18, 19], based on the inverse relationship between the effective population size (N_e_) [20] and the rate of erosion of genetic variation by drift, which is supported by studies of wild populations [21, 22]. N_e_ estimates tend to be low in relation to census population size in natural populations [23, 24], influenced by demographic fluctuation and life-history traits [25, 26]. Both small population size and genetic erosion render populations more susceptible to stochastic environmental and demographic adverse events, such as climate change or disease [19, 27, 28]. Small populations isolated by habitat fragmentation are also at increased risk of inbreeding. However, several important questions regarding genetic variation and inbreeding depression in natural populations remain largely unanswered. In particular, it is unclear to what extent mating between close relatives and loss of genetic diversity contribute to population decline and extinction in the wild, and thus to how results of genetic studies should influence their conservation management [29].

We report the results of the UK Adder Genetic Project (UKAGP), a study into the genetic status of lowland adder populations in southern mainland Britain, where national distribution surveys have indicated a decline in comparison with historic records [30–33]. A national questionnaire-based investigation survey of adder populations showed that declines were more likely to be reported in small sites with fewer than ten adders, whether based on systematic surveys or anecdotal evidence [31]. The subsequent Make the Adder Count (MTAC) initiative, based on peak springtime adder counts over sequential years, further underscored the increased risk of decline in small populations [34], flagging threats of habitat loss, public disturbance and predation, especially by cats and birds. To these threats should be added the potential risk of disease caused by the release of captive non-native snakes onto adder habitat, especially in view of the recent finding of the causative agent for snake fungal disease (*Ophidiomyces ophiodiicola*) in UK adders in the wild [35]. This consistently emerging picture of habitat fragmentation and local decline forms the background for our study, in which we have used a combination of mitochondrial DNA (mtDNA) and microsatellite markers to investigate the potential role of genetic factors in their decline of adders in mainland Britain.

The aim of this study was to document population genetic structure and differentiation, and to estimate indicators of inbreeding and genetic diversity in lowland adder populations in southern mainland Britain. To assess the ecological and conservation significance of our study, we interpret our results in comparison with published studies of adder populations in mainland Europe, and with reference to the size, and thus the likely risk status, of the study populations.

## Methods

### UKAGP study sites and samples

Ethics and animal welfare: the project was reviewed prospectively and approved by the ZSL Ethics Committee. Sampling was undertaken in concordance with ZSL ethical guidelines. Cloacal swabs were collected without anaesthetic from adult snakes, and buccal swabs from juveniles [36], by ecologists experienced in snake handling. All snakes were released at the site of capture. Handling of adult females was avoided after mid-May, to minimise disturbance to gravid snakes. No study animal was subjected to euthanasia.

Samples were collected from 220 adders at 16 sites in southern mainland Britain between March and May 2011 (Table 1). No permits were required, as there were no restrictions on site access, and the adder in the UK has no specific protection status other than against deliberate injury or killing, or collection for trade (Wildlife and Countryside Act 1981, as amended 1991). For each site, adders were caught over a one to two-day period. Dates and optimal weather conditions for sampling were determined according to local ecological expertise.

**Table 1:** Details of sites and samples.

Site		Lat	Long	Alt	Size	Total (young)	Male	Female	mtDNA	msat
Woodbury Common	WC	50.68 N	3.37 W	174	large	15 (3)	2	13	13	11
Blackmoor Reserve	BM	51.30 N	2.71 W	247	large	26	15	11	12	20
Cranham Common	CC	51.81 N	2.15 W	200	small	4	0	4	4	4
Crickley Hill	CH	51.82 N	2.12 W	274	small	10	7	3	6	10
Ewyas Harold	EH	51.96 N	2.89 W	124	large	11	4	7	7	10
Malvern Hills Swinyard	MHS	52.09 N	2.34 W	251	large	20	12	8	5	17
Bradnor Hill	BH	52.22 N	3.06 W	292	small	5	1	4	5	7
Bircher Common	BC	52.30 N	2.78 W	199	small	9	8	1	6	6
Mortimer Forest	MF	52.36 N	2.74 W	87	large	18 (1)	12	6	6	13
Wyre Forest	WF	52.41 N	2.32 W	139	large	19	18	1	5	16
Pounds Green Coppice	PGC	52.41 N	2.36 W	97	small	7	4	3	5	6
Kinver Edge	KE	52.44 N	2.25 W	143	small	6	4	2	4	6
Thundry Meadow	TM	51.19 N	0.72 W	55	small	5	2	3	3	5
Holt Lowes	HL	52.89 N	1.10 E	66	large	27	24	3	5	20
Martlesham Heath	MH	52.06 N	1.26 E	27	small	9 (1)	7	2	6	9
Dunwich	DUN	52.26 N	1.62 E	18	large	25	28	1	5	25

The total number of samples for each site is given; the number of samples from young adders (juveniles or subadults) is shown in brackets (included in total).

Lat/long: point GPS coordinates latitude and longitude for site (not for sampling of individual adders); Alt: altitude (metres above sea level) derived from google map for site coordinates.

mtDNA: number of individuals genotyped for mtDNA; msat: number of samples genotyped for microsatellites at minimum of 6 of 8 loci.

size: allocation to MTAC category [34], according to peak springtime time count > 10 = large, ≤ 10 = small.

DNA was extracted using a DNeasy blood and tissue kit (Qiagen), following the manufacturer’s protocol for swabs.

### Mitochondrial DNA (mtDNA) sequencing

We designed primers from a 918-bp mtDNA control region (CR) sequence and a 1043-bp mtDNA cytochrome b sequence (Cytb) [37], based on high levels of variability across 40 European adder haplotypes, and on consistent flanking region stability. The primer sequences selected for this study are shown in Table 2A. PCR for both loci was performed in HotStarTaqPlus (Qiagen), with an annealing temperature of 55°C. The same primers were used for sequencing reactions. PCR products were cleaned using a QIAquick PCR purification kit (Qiagen, UK), and sequenced using ABI BigDye® chemistry and 3130XL sequencer, following manufacturers’ protocols. We generated alignments of concatenated Cytb/CR consensus sequences using MEGA 6 [38]. Individual haplotypes were identified using the *haplotype* function of pegas package v0.10 [39], implemented in R v3.4.0 [40]. A haplotype network [41] was constructed using the median‐joining method [42] in NETWORK v4.6.1 (www.fluxus-engineering.com). Phylogenetic analysis was carried out as described in Supporting Information (S1 Table, S1 Fig).

**Table 2:** Primers used in mtDNA PCR and sequencing.

Locus	Primer	Amplicon
CR	5’-TGC CCC ATG GAT ATT AAG CCG GA-3’	349 bp
	5’-AAC CAG CGG CCT TGG AAA GGA -3’	
Cytb	5’-CCA AAC CAT TAC TGG ATT CTT CC-3’	265 bp
	5’-ATA GCC GAA GAA GGC TGT TGC-3’	

Primers were designed for this study as described in methods, based on original sequences from Ursenbacher et al (2006) [37]. Amplicon: size of PCR product for each locus.

### Microsatellite genotyping

In preliminary studies we tested published microsatellite primers that had been developed for adders (*V*. *berus*) [43, 44], selecting five polymorphic loci that we found to amplify consistently, with a minimum of stutter bands. To increase the number of loci, we also evaluated 15 congeneric microsatellite markers which had been developed for meadow vipers (*V*. *ursinii*) [45], selecting three that successfully amplified and demonstrated polymorphism in adder samples (results not shown). PCR was performed in 10 μl volumes with 20–100 ng DNA, 5 μl mastermix (HotStarTaq Plus or Multiplex; Qiagen), 5 μmol/L unlabelled reverse primer and 5 μmol/L fluorophore-labelled forward primer (Applied Biosystems). Amplification was performed in simplex with initial denaturation 95°C 5 min, 60 cycles of 94°C 60 sec, 57–59°C 60 sec, 72°C 60 sec, and final extension 72°C 7 min, optimized in preliminary studies for each primer pair. Primer sequences and locus-specific PCR conditions are summarized in Table 3. Amplified products were resolved by capillary electrophoresis on a 3130xl Genetic Analyser with a LIZ-500 size standard (Applied Biosystems). Alleles were scored and binned manually, using PeakScanner 1.0 software (Applied Biosystems).

**Table 3:** Microsatellite loci: primers and PCR conditions.

Locus	Ref	Origin	Repeat	PCR	n^o^ alleles
**Vu57**	a	V.ursinii	2	60 MP	8
**Vu4**	a	V.ursinii	3	60 HS	11
**CA71**	c	V.berus	2	60 HS	11
**Vb-B’2**	b	V.berus	2	58 HS	24
**CA11**	c	V.berus	2	60 MP	31
**CA3**	c	V.berus	2	58 HS	19
**Vb-B’10**	b	V.berus	2	55 MP	9
**Vu18**	a	V.ursinii	2	55 MP	9

origin: species for which locus had been developed.

PCR: annealing temperature °C and PCR buffer system.

MP: Multiplex mastermix (Qiagen), HS HotStarTaqPlus mastermix (Qiagen).

repeat: size of microsatellite repeat motif in nucleotides.

n^o^ alleles: number of alleles for relevant locus in total study dataset.

ref: a) Metzger et al (2011) [45]; b) Ursenbacher et al (2009) [44]; c) Carlsson et al (2003) [43].

### Microsatellite data analysis

#### Quality control

Replicates and template negative controls were included in all plates to confirm reproducibility of results. Results were analysed for genotyping errors and null alleles in Micro-Checker v 2.2.0.3 [46]. We used FSTAT v2.9.3.2 [47] and pegas [39], implemented in R, to test for Hardy-Weinberg equilibrium (HWE), and to exclude linkage disequilibrium.

#### Measures of population structure and differentiation

In this study, we use the term population to refer to all individuals sampled at a single study site in the sampling time period. Pairwise F_ST_ values between populations were estimated in FSTAT.

To test for isolation by distance [48] we applied the *mantel*.*rtest* function of ade4 v1.7–13 [49], implemented in R, with 999 repetitions, using pairwise F_ST_ to estimate genetic distance. Geographic distance was estimated at https://andrew.hedges.name/experiments/haversine (accessed 23 April 2108) using the Haversine great circle method, a measure of the shortest distance between two points on a sphere [50].

To investigate genetic differentiation, we used STRUCTURE v2.3 [51, 52], using correlated allele frequencies and admixture models, with or without the *locprior* option [53]. The initial alpha was set at 1/n, where n is the number of sample locations, to allow for variation in sample sizes between populations [54]. We used burn-in of 10^5^, followed by 10^6^ iterations for 10 independent replicate runs for values of K from 1 to the number of populations being studied. Results were uploaded to StructureHarvester [55] to derive mean log likelihood and delta-K as a function of K, detecting hierarchical levels of structure [56]. Results across replicate runs were permuted using the *greedy* function of CLUMPP [57] to derive proportional assignments to each cluster for supported values of K. We also studied population structure using discriminant analysis of principal components (DAPC) [58] in adegenet version 2.0.1 [59] implemented in R. DAPC is a multivariate method to identify clusters of genetically related individuals, which is not based on a predefined model, and makes no assumptions of HWE. The *find*.*clusters* function was applied to determine the optimal number of clusters (k) in each population, independent of the number of sampling sites. The *dapc* function was then applied, using the α- score function to determine the optimum number of principal components to retain in each analysis. Probabilities of assignment of individuals to each of the different clusters were visualised using the *compoplot* function of adegenet [59].

#### Microsatellite summary statistics: Baseline indicators of genetic diversity and inbreeding

For each population, we estimated F-statistics [60] and allele richness in FSTAT. Confidence intervals for F_IS_, a measure of intrapopulation heterozygote deficiency due to inbreeding, were calculated using the *boot*.*ppfis* function of hierfstat package v0.04–22 in R [61]. Mean allele richness was determined using a rarefaction method [62] in PopGenReport [63], implemented in R. We used FSTAT to compare populations with respect to allele richness and F-statistics, using 1000 permutations.

#### Detection of population bottlenecks

We used BOTTLENECK v 1.2.02 to test for significant heterozygosity excess, applying a one-tailed Wilcoxon test with 1000 iterations, using the two-phase model (TPM) (90% stepwise mutations, variance 10) [64]. A mode-shift test for distortion of the allele frequency distribution [65] was also implemented in BOTTLENECK. We derived M-ratio statistics [66] using the *mRatio* function of the strataG package v 2.0.2 [67], implemented in R.

#### Effective population size (N_e_)

Two single sample methods were used for estimation of N_e_. The linkage disequilibrium method [68, 69] was implemented in NeEstimator ver 2.1 [70], assuming random mating, deriving confidence intervals by jack-knifing (1000 iterations). We also used the sibship assignment method [71], which estimates the current effective breeding size of the population, implemented in COLONY 2.0.6.3 [72], using the same input parameters as detailed below for sibship and parentage analysis. Confidence intervals were obtained by bootstrapping.

#### Further investigation of breeding between relatives

We applied the *inbreeding* function of adegenet, version 2.0.1 [59] in R, to derive genetic estimates of the pedigree inbreeding coefficient FPED, which denotes the probability that both alleles at a single locus are identical by descent from a single ancestor [73]. Pairwise relatedness (Rxy) was estimated using a maximum likelihood method in ML-Relate [74]. To calibrate Rxy values with first degree family relationships in our data, we simulated genotypes for pairs of individuals with defined relationships (100 pairs for each category of unrelated, half-sibling, full-sibling and parent-offspring), using the *familysim* function of the package related v1.0 [75], implemented in R, based on allele frequency data of observed datasets. Pairwise Rxy between each pair of simulated genotypes of defined relationships was measured using ML-Relate. Means and confidence intervals were derived in R. Significance of within-population relatedness was tested using the *grouprel* function of related v1.0.

For sibship and parentage analysis we used a full-likelihood method, implemented in COLONY 2.0.6.4 [72], assuming both male and female polygamy. In the absence of known pedigree structure, all individuals for each study population were treated as a single offspring group. We used default settings for sibship priors, including small sibship size, with the aim of reducing false sibship assignments [76]. The outputs of three independent replicate runs, using independent seeds for random number generation, were examined to confirm convergence to the same configuration and log likelihood. The best maximum likelihood cluster configuration was used to infer half- and full sib dyads and inferred parentage.

#### Interpretation of results

We compared our results for F-statistics and allele richness from UKAGP study populations with summary statistics from two published studies of adder populations in mainland Europe [44, 77], and from a site containing a very large (n >500) population of lowland adders in northern Belgium ([78], Mergeay & Bauwens unpublished data). Direct statistical comparison was precluded by only partially overlapping microsatellite panels between the studies (2/8 loci of our study were in common with Ursenbacher et al [44, 77]; 3/8 loci in common with Bauwens et al [78]).

In the UK, the MTAC survey demonstrated opposite average population trends between sites with small and large adder populations, the threshold being a mean normalised peak count of 10 adders, below which there was significant decline over time [34]. This provides an approach of demonstrated demographic relevance with which to classify and compare populations according to their likely risk of decline. At eight sites in our study, more than 10 individuals had been sampled on a single visit (range 11–29). The number of adders sampled at the other eight sites was lower (range 4–10) (Table 1), despite equivalent or higher sampling effort by an experienced ecologist familiar with the sites. We therefore applied an equivalent threshold to categorise UKAGP study populations as large (presumed lower risk) (count >10, n = 8), or small (presumed higher risk) (count ≤10, n = 8) (Table 1), based on the number of adders sampled, as an approximation of the MTAC criteria. We compared first-line summary statistics, FPED and R_xy_ between small and large populations defined in this way, using a Wilcoxon rank sum test implemented in R.

Although exceeding the MTAC peak count threshold, the springtime counts for the UKAGP populations in the “large” category were still relatively low, with a maximum of 27. In the absence of a very large well-characterised UK population for comparison, we analysed 50 genotypes from the Belgian site [78], focusing primarily on indices of relatedness and inbreeding. This 1570 ha site (“Groot Schietveld”, N 51° 20–22’–E 4° 32–37’), has been used as a military exercise zone since 1893, and is separated from neighbouring adder populations by a minimum of 18km of unsuitable agricultural habitat. The site is transected by a road, constructed in 1875 as a narrow cobble road, then transformed in 1982 to its present state as a two-lane asphalt trunk road, flanked by two asphalt cycle tracks (total width ca. 20 m). In capture-mark-recapture studies, the road was shown to constitute an effective hard barrier to adder migration (Claus & Bauwens, unpublished data). The sample of 50 Groot Schietveld genotypes (GS50) was selected randomly from the total dataset of microsatellite genotypes of individual adders, which had been sampled from 14 locations within the Groot Schietveld site between 2011 and 2013, and genotyped for 9 microsatellite loci as detailed in Bauwens et al 2018 [78]. The sample included 18 individuals from the north east (NE) segment of the site, and 32 from the south west (SW), relative to the transecting road. No mtDNA sequence data were available for the GS50 sample.

## Results

### mtDNA haplotypes

**Fig 1:**
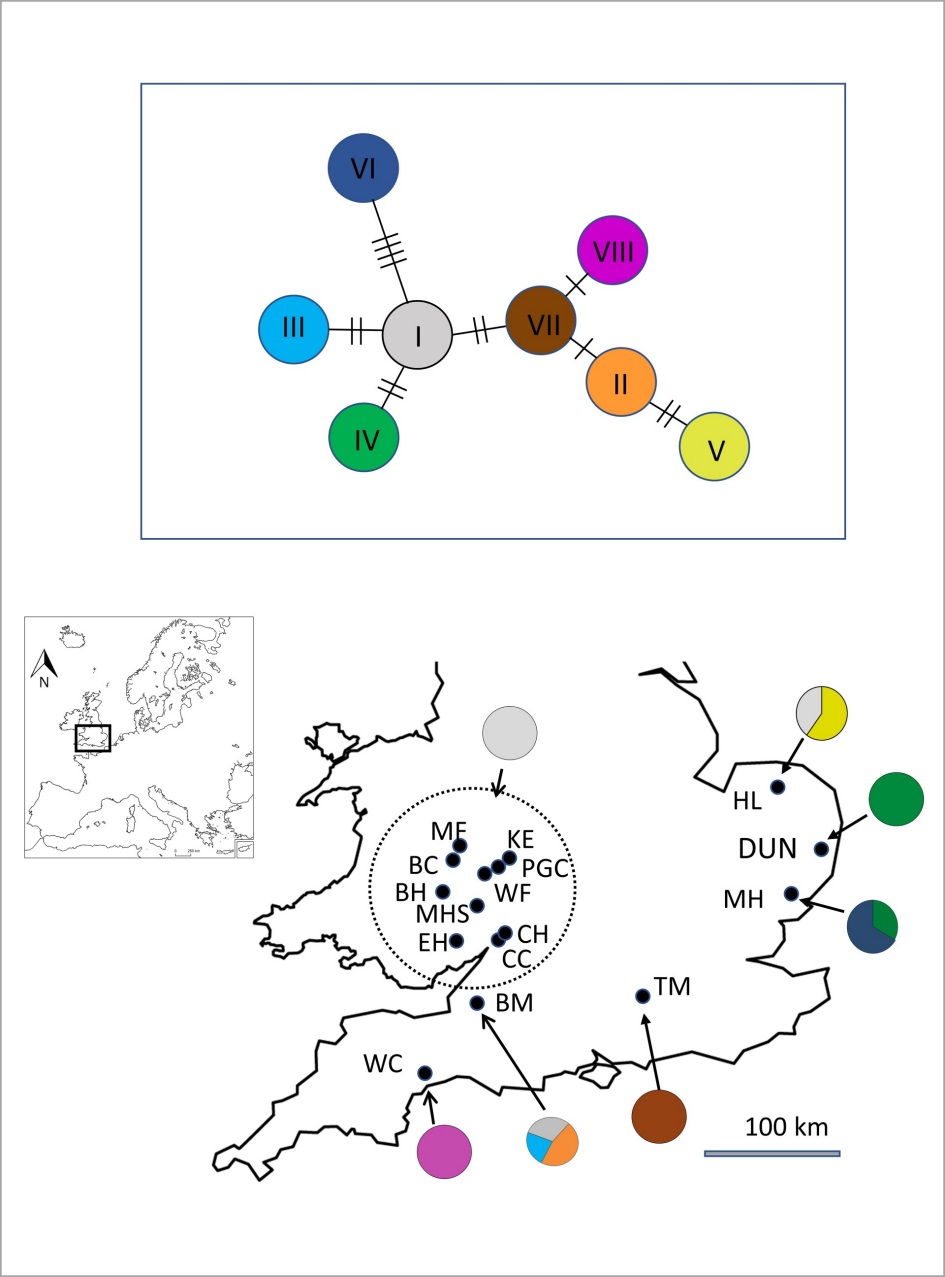
Location of study sites and mtDNA haplotypes. Outline map of southern Britain showing approximate locations of study sites. Site abbreviations and coordinates are detailed in Table 1. Pie charts illustrate the distribution of the mtDNA haplotypes shown in the mtDNA CytB/CR haplotype network (top). Lines denote nucleotide differences between haplotypes. The sites sharing the same haplotype (WMids Haplogroup) are circled.

Eight different Cytb/CR haplotypes were identified in 97 individuals across the 16 sites (Fig 1). Six haplotypes were unique to single sites. Three sites had more than one Cytb/CR haplotype. All 53 individual samples sequenced from a cluster of ten sites in the West Midlands/South West had the same Cytb/CR haplotype (WMids Haplogroup). As predicted, the UKAGP haplotypes broadly clustered with the mainland Europe Northern phylogenetic clade of *V*.*berus*, as described by Ursenbacher et al 2006 [37] (S1 Fig).

### Microsatellites

#### Quality control

There was no evidence for null alleles, allele drop out or linkage disequilibrium between loci. No two samples had identical genotypes. Results from duplicate samples confirmed consistency of genotyping. Samples that failed to amplify at a minimum of 6/8 microsatellite loci were excluded from analysis. In all, 186 samples (84.5%) were retained for downstream analysis. There was no significant divergence from HWE. Two small populations TM (n = 5) and CC (n = 4) had missing data for more than one individual at a single locus (CA71 and Vu4 respectively). For these, genetic diversity was calculated with the omission of the relevant locus, which had little impact on summary statistics for the other populations (S2 Table).

#### Genetic substructure of WMids Haplogroup

The WMids Haplogroup includes ten geographically neighbouring sites, separated by up to 100km (Fig 1, Table 4). All individuals tested in these sites shared the same mtDNA haplotype, consistent with their origin from a common ancestor. We therefore investigated this group for evidence of more recent differentiation, using microsatellite markers. Although this haplotype was found two in other study sites, they were not included in this analysis, as both showed evidence for additional haplotypes, and were from less intensively sampled regions, with larger distances between sites.

**Table 4:** Genetic differentiation between populations in WMids Haplogroup.

	EH	CH	KE	MF	WF	PGC	MHS	BC	BH
**EH** (11)		55	69	45	66	61	43	39	32
**CH** (10)	0.113		107	106	103	104	74	102	105
**KE** (6)	0.153	0.079		36	4	9	35	40	60
**MF** (13)	0.105	0.085	0.03		35	28	38	6	24
**WF** (16)	0.1	0.049	0.071	0.055		8	31	38	58
**PGC** (6)	0.167	0.082	0.14	0.11	0.082		30	31	51
**MHS** (17)	0.155	0.164	0.169	0.119	0.104	0.136		35	50
**BC** (6)	0.155	0.13	0.142	0.09	0.109	0.188	0.108		20
**BH** (7)	0.15	0.098	0.101	0.052	0.096	0.109	0.168	0.129	

Pairwise FST (genetic distance) below diagonal, geographic distance (km) above diagonal. Sample sizes are shown in brackets.

Shaded values are significant at p <0.05 level after 12000 permutations, with Bonferroni correction.

Site abbreviations as in Table 1, geographic locations as in Fig 1. Site CC is excluded, due to its small size and missing data.

**Fig 2:**
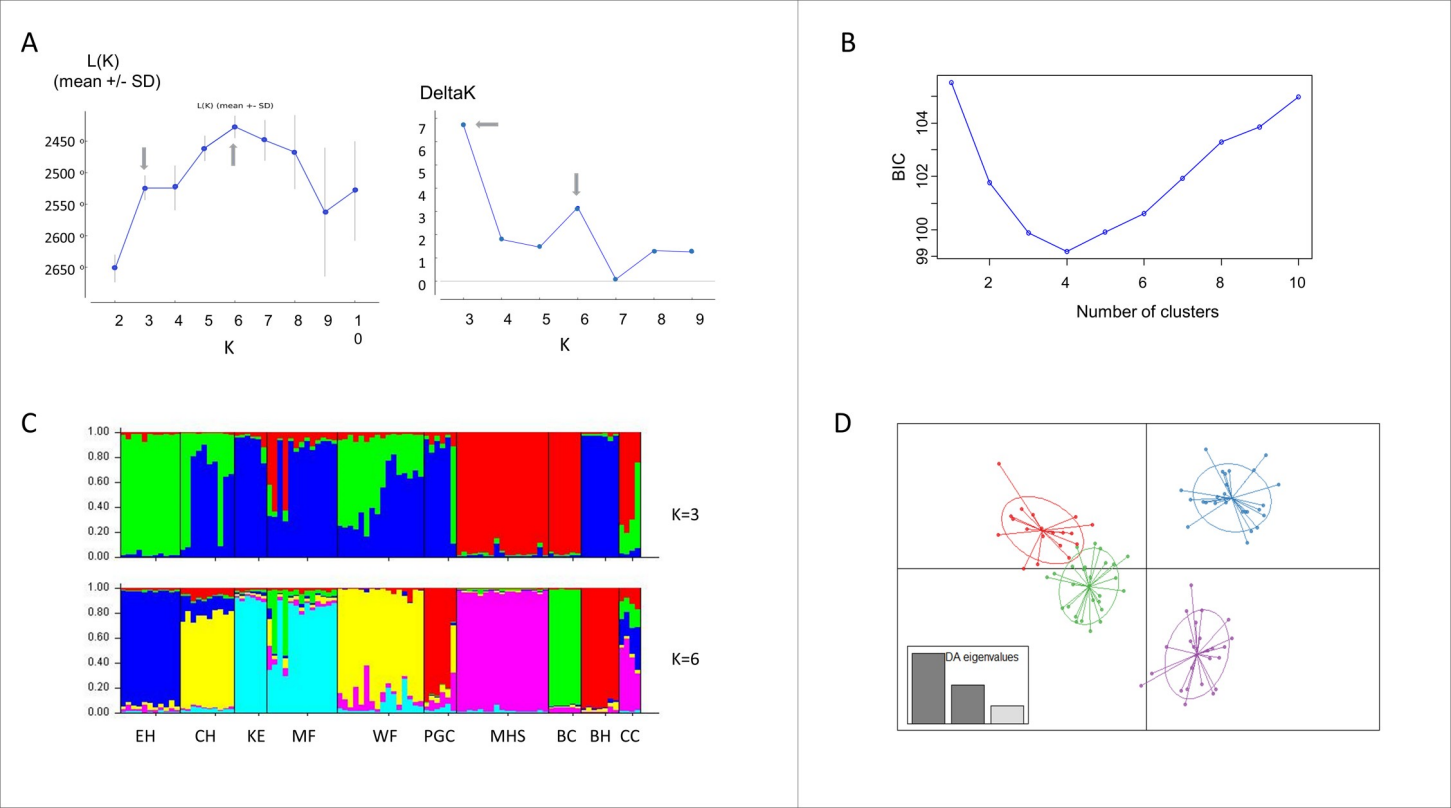
Hierarchical genetic substructure of the WMids Haplogroup. (**A**) STRUCTURE: graphs for posterior probability L(K) and deltaK showing highest probability for K = 3 and K = 6 (with *locprior* option). (**B**) DAPC: graph of BIC, showing a clear elbow at K = 4. (**C**) STRUCTURE: bar charts showing proportional membership coefficients of individuals to each of the inferred clusters for K = 3 and K = 6, grouped according to their study population. Each colour represents a different cluster. (**D**) DAPC: scatterplots of individuals and inertia ellipses in four clusters, defined according to the clustering algorithm in adegenet.

Genetic differentiation within the WMids Haplogroup is reflected in the pairwise F_ST_ matrix between these sites (Table 4). Mantel testing for isolation by distance was negative (r = 0.0042; simulated p value = 0.538). In STRUCTURE there was support for hierarchical clustering [56], both with and without applying the *locprior* option (Fig 2; S2 Fig). DAPC analysis also showed clustering within the WMids Haplogroup, optimal at four clusters (Fig 2). To evaluate concordance between STRUCTURE and DAPC results we also applied the *dapc* function for three and six clusters, the optimum values for K in STRUCTURE using the *locprior* option. The population proportional assignments to each cluster by each of the two methods was very similar at the higher hierarchical level of clustering (K = 3), but more divergent for K = 6 (S3 Fig).

#### Genetic diversity and inbreeding (F_IS_)

F_IS_ values did not differ significantly from zero in any of the UK study populations (Table 5). This is in line with the findings of the mainland Europe study, where only 2/16 sites had been reported to have raised levels of F_IS_, in both cases attributable to high homozygosity at a single locus [44]. Estimates of genetic diversity (H_S_ or H_E_) were at similar levels across the study sites, and broadly equivalent to published results from European populations [44, 77], including the large Belgium lowland population [78] (Table 6). Allele richness was in a similar range to that of the large lowland Belgian population, and of the Belgian, Netherlands and northern France lowland populations in the studies of Ursenbacher et al [44, 77], although with the caveat of only partially overlapping microsatellite panels. We found mean allelic richness was lower (p<0.05) in small populations. This was the only statistic for which small and large populations differed significantly, other than for size (Table 7).

**Table 5:** Summary statistics for individual study populations.

	n	Ar	Ho	Hs	FIS	FST
**EH**	11	2.42	0.63	0.64	0.02	0.03
**CH**	10	2.75	0.70	0.73	0.05	-0.03
**WC**	11	2.79	0.79	0.73	-0.08	0.04
**KE**	6	2.67	0.67	0.76	0.11	-0.10
**MF**	13	2.73	0.66	0.73	0.09	0.02
**BM**	20	2.61	0.71	0.69	-0.03	0.00
**WF**	16	2.75	0.75	0.73	-0.02	0.04
**MH**	9	2.60	0.61	0.69	0.12	0.03
**PGC**	6	2.61	0.65	0.72	0.10	-0.02
**DUN**	25	2.60	0.70	0.69	-0.02	0.00
**HL**	20	2.72	0.73	0.72	-0.02	0.01
**MHS**	17	2.60	0.62	0.69	0.10	-0.03
**BC**	6	2.43	0.65	0.67	0.04	-0.03
**BH**	7	2.61	0.77	0.68	-0.13	0.08
TM*	5	1.99	0.46	0.50	0.09	0.16
CC**	4	2.36	0.68	0.59	-0.15	0.06

no: number of samples genotyped at a minimum of 6/8 loci.

TM*: 7 loci, excluding CA71; CC**: 7 loci excluding Vu4.

Ar: allele richness; Ho: observed heterozygosity; Hs: gene diversity (expected heterozygosity); n: sample size.

site abbreviations as in Table 1.

**Table 6:** Comparison of mean summary statistics between UK study and sites from mainland Europe.

UK AGP	no	Ar	Ho	Hs	FIS	FST	Comment
**mean**	11.63	2.58	0.67	0.69	0.02	0.02	current study
**SE**	1.58	0.05	0.02	0.02	0.02	0.01	16 pops; lowland
**Ursenbacher 2009 [44]**	**no**	**Ar**	**Ho**	**He**	**FIS**	**FST**	
**mean**	24.50	2.98	0.50	0.52	0.01		Mainland Europe
**SE**	4.09	0.02	0.02	0.14	0.02		16 pops; montane
**Ursenbacher 2015 [77]**	**no**	**Ar**	**Ho**	**He**	**FIS**	**FST**	
**MC mean**	16.60	3.62		0.61		0.26	Massif Central
**SE**	1.00	0.13		0.02		0.01	10 pops; montane
**JM mean**	23.67	2.70		0.44		0.36	Jura Mountains
**SE**	2.32	0.26		0.05		0.03	6 pops; montane
**AC mean**	18.33	4.06		0.67		0.23	Atlantic Coast
**SE**	0.88	0.01		0.01		0.01	3 pops; lowland
**NE mean**	17.56	2.46		0.39		0.40	NE France, Belgium, NL
**SE**	2.29	0.19		0.05		0.03	9 pops; lowland
**Bauwens 2018 [78]**	**no**	**NeA**	**Ho**	**He**	**F**	**FST**	
**mean**	599.00	2.50	0.52	0.55	0.08		Belgium
**SE**	na	0.26	0.07	0.07	0.03		single pop; lowland

Means and standard errors (SE) for equivalent summary statistics between groups of populations from different studies, except Bauwens 2018, where results are from a single large population.

Shaded cells denote no comparable information available.

NeA = number of effective alleles; Ho: observed heterozygosity; Hs: gene diversity (expected heterozygosity); F = fixation index.

MC: Massif Central; JR: Jura Mountains; AC: Atlantic coast; NE: north east France, Belgium and Netherlands (NL); na: not applicable.

**Table 7:** Comparison of results between small and large study populations.

	Size		mAr		Ar		Hs (He)		Ho		Ho:He		FIS		FST		Rxy		F_PED_	
	Small	Large	Small	Large	Small	Large	Small	Large	Small	Large	Small	Large	Small	Large	Small	Large	Small	Large	Small	Large
**min**	4	11	2.45	2.74	1.99	2.42	0.50	0.64	0.46	0.62	0.88	0.90	-0.15	-0.08	-0.10	-0.03	0.17	0.14	0.18	0.19
**max**	10	25	2.97	3.03	2.67	2.79	0.76	0.73	0.77	0.79	1.15	1.08	0.12	0.10	0.16	0.01	0.38	0.27	0.28	0.27
**mean**	6.14	15.89	2.72	2.92	2.47	2.66	0.66	0.71	0.64	0.70	0.97	0.99	0.03	0.01	0.03	0.01	0.25	0.19	0.24	0.23
**SE**	0.72	1.74	0.05	0.04	0.04	0.04	0.01	0.01	0.02	0.02	0.04	0.02	0.04	0.02	0.03	0.01	0.02	0.02	0.01	0.01
**wcox p**	<0.001		<0.05		0.09 ns		0.24 ns		0.17 ns		0.35 ns		0.41 ns		0.53 ns		0.10 ns		0.54 ns	

Comparison of results between the eight small and eight large study populations (as detailed in Table 1).

mAR: mean allele richness; Ar: allele richness; Ho: observed heterozygosity; Hs: gene diversity; He: expected heterozygosity.

Rxy pairwise relatedness within population; F_PED_: genetic estimate of inbreeding coefficient.

wcox p: p value from comparison between small and large using Wilcoxon test in R; ns: not significant (p value >0.05).

#### Effective population size N_e_

The single sample LDNe method [69] failed to deliver plausible results, which may reflect small sample size and high levels of relatedness [79]. Results derived using the sibship assignment method are shown in Table 8. The small populations again gave very wide confidence intervals. Results for GS50 SW and NE by the sibship assignment method are also shown, generating lower results than expected for the very large number of adders on the site, discussed further below. Unfortunately, the combination of small sample size and high relatedness thus prevented us from deriving reliable estimates for effective population size, a significant disadvantage in the study of wild populations.

**Table 8:** Estimate of effective population size by sibship assignment method.

		Assuming random mating			Assuming non-random mating			
	n	Ne	CI 95(L)	CI 95 (H)	Ne	CI 95(L)	CI 95 (H)	alpha
**all**	186	186	150	240	120	93	154	0.19
**WMHg**	96	86	62	116	53	37	80	0.2
**BC**	6	15	6	>>>	12	5	>>>	0.08
**BH**	7	7	3	26	7	2	30	0.01
**BM**	20	14	7	33	11	5	28	0.12
**CC**	4	8	2	>>>	19	6	>>>	-0.19
**CH**	10	4	8	25	6	2	21	0.13
**DUN**	25	18	9	36	13	7	31	0.15
**EH**	11	10	5	30	7	3	28	0.14
**HL**	20	25	14	30	19	11	42	0.09
**KE**	6	9	4	96	7	2	487	0.08
**MF**	13	12	6	30	8	3	28	0.16
**MH**	9	16	7	64	10	4	43	0.2
**MHS**	17	16	8	35	10	5	27	0.22
**PGC**	6	9	4	56	6	2	49	0.13
**TM**	5	40	8	>>>	26	5	>>>	0.17
**WC**	11	10	5	28	9	4	26	0.03
**WF**	16	22	11	54	17	8	41	0.1
**GS50 SW**	32	14	8	30	12	6	26	0.07
**GS50 NE**	18	12	6	30	9	4	24	0.12

Effective population size using sibship assignment full likelihood method, with 95% confidence intervals.

all: entire UKAGP dataset; WMHg: WMids Haplogroup; n: sample size; >>>: > 10^9.

#### Population bottlenecks

As small sample size may give rise to false positives in bottleneck tests, we tested different sample sizes randomly selected from the simulated population of 100 unrelated pairs. A sample of 5 genotypes generated a positive result for both heterozygosity excess and modal shift. Results for both tests were negative for simulated samples of 10 or 20 (Table 9). In two of the UKAGP study populations with a sample size of ≥10, both heterozygosity excess and allele frequency modal shift tests generated positive bottleneck results (EH, WF). Two (BM, CH) was positive for modal shift only. MRatio results did not discriminate between any of the simulated or study populations, irrespective of size, or results of other bottleneck tests (Table 9).

**Table 9:** Testing for population bottlenecks.

Individual		Heterozygote excess				Mratio	
Populations	Number	TPM 10/90	SMM	Mode shift	Mean	95% L	95% H
CH	10	0.32	0.37	shift	0.43	0.26	1.00
EH	11	0.03	0.19	shift	0.63	0.25	0.99
MF	13	0.27	0.32	normal	0.43	0.20	0.66
MHS	17	0.37	0.77	normal	0.53	0.29	0.77
BM	20	0.16	0.27	shift	0.49	0.27	0.71
WC	11	0.32	0.42	normal	0.48	0.27	0.69
DUN	25	0.32	0.42	normal	0.43	0.27	0.60
HL	20	0.63	0.84	normal	0.42	0.27	0.57
WF	16	0.00	0.01	shift	0.44	0.30	0.58
**simulated dataset**							
sim UR 200	200	0.77	0.99	normal	0.44	0.23	0.74
sim UR 20	20	0.41	0.81	normal	0.49	0.38	0.61
sim UR 10	10	0.53	0.68	normal	0.36	0.20	0.61
sim UR 5	5	0.10	0.27	shift	0.32	0.16	0.51

Top panel: results of tests using three methods (heterozygote excess, mode shift of allele frequency distribution curve, and Mratio) for populations with sample size ≥10. Positive results are shaded.

TPM: two phase model; SMM: stepwise mutation model (see methods for details). Results for heterozygote excess are shown as p values (one-tailed Wilcoxon, 1000 iterations).

Bottom panel: results of tests applied to different sample sizes of simulated populations, comprising individual genotypes drawn randomly from 100 simulated unrelated pairs, based on the total UKAGP dataset (for which bottleneck tests were negative with all methods), showing false positive results for sample size of 5.

#### Further investigation of breeding between relatives

**Fig 3:**
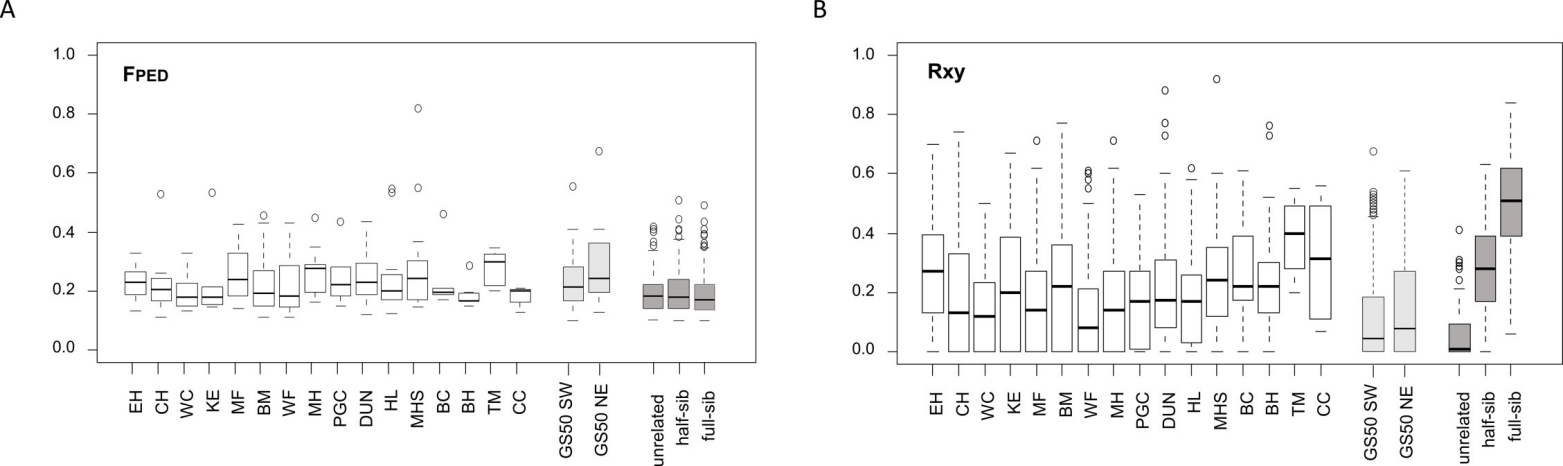
Inbreeding and pairwise relatedness. Box and whisker plots of genetic estimates of FPED (**A**), and for pairwise relatedness Rxy (**B**) for the study populations (open box) GS50 SW and NE (light shading), and for 100 simulated pairs of genotypes of defined relationship (dark shading). Individual outliers are shown as open circles.

Estimated inbreeding coefficient FPED results showed little variation between populations, irrespective of size (Table 7). The mean population FPED was 0.233 (95% CI 0.218–0.248), although some populations had individual outliers with FPED >0.50 (Fig 3). We found a similar pattern of FPED results for the GS50 sample, again with occasional outliers FPED >0.50. FPED results did not differentiate between simulated populations of 100 pairs of defined relationship, whether unrelated, half- or full sibling (Fig 3).

**Fig 4:**
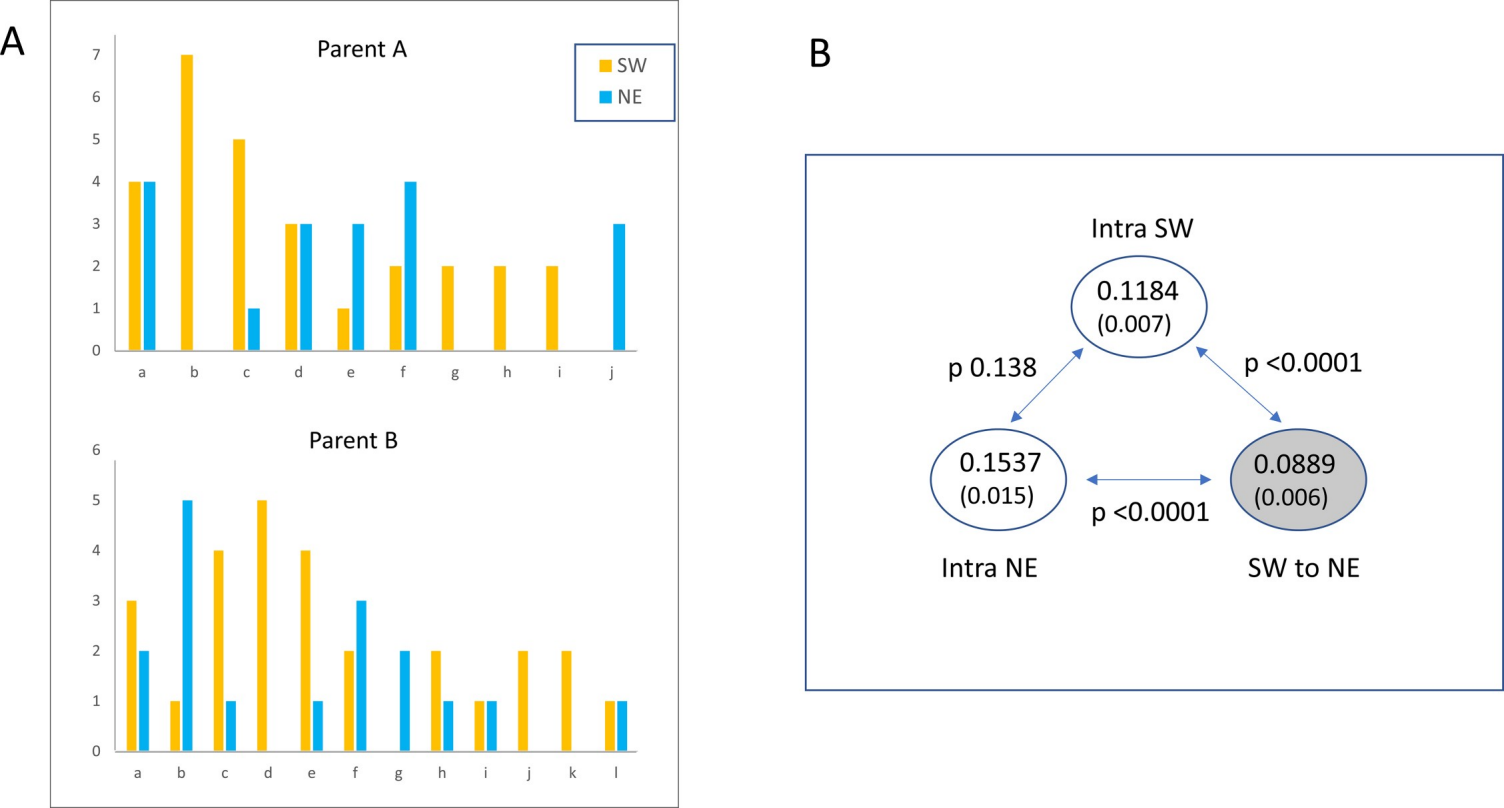
GS50: Dividing road acts as barrier to gene flow. (**A**) Bar chart of inferred parentage of individuals of GS50 sampled from NE and SW of the transecting road. The best-supported cluster in COLONY for the full GS50 sample inferred a total of 10 parents of one sex (parent A), and 12 of the other sex (parent B). The bar charts show the number of individuals (“offspring”) from the NE (blue) and SW (orange) assigned to each inferred parent. The difference in inferred parentage is in keeping with the road acting as a barrier to gene flow. (**B**) Significantly lower mean pairwise Rxy sampled from different sides of the dividing road (SW to NE (shaded)) than between individuals sampled from the same side of the road (intra SW, intra NE), again consistent with the road acting as barrier to gene flow.

Intra-population mean Rxy estimates ranged from 0.135 to 0.377 (mean 0.220, 95% CI 0.188–0.252), with no significant difference between large and small populations (Fig 3; Table 7). All UKAGP study populations showed significant within-population relatedness (S3 Table). For the GS50 sample, the mean Rxy between individual samples from the same side of the transecting road (intra-SW, intra-NE) was significantly higher than for pairwise Rxy across the road (SW-NE) (Fig 4). As expected, and in contrast to FPED, mean Rxy differed significantly between simulated pairs of defined relationship (Fig 3).

**Fig 5:**
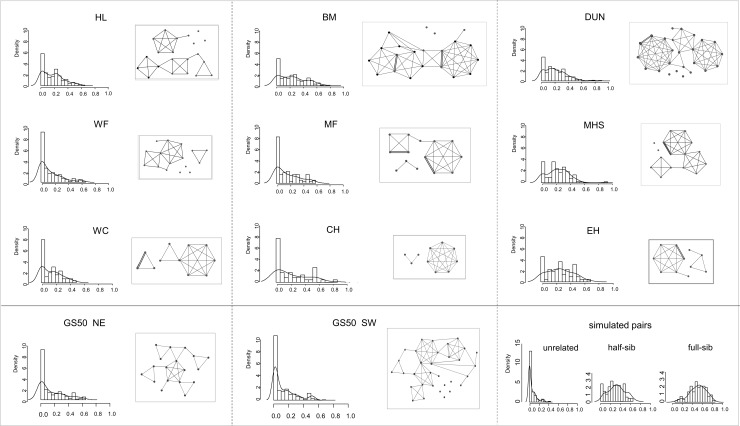
Networks of inferred sibships and frequency distribution curves of pairwise relatedness. Network of related individuals at study sites with sample size ≥10 and for GS50 NE and SW subsamples, according to best maximum likelihood configuration in COLONY. Individuals are shown as a filled circle. Inferred half-sib dyads are linked by single lines, full-sib dyads by double lines. Individuals with no inferred first-degree relationships are shown as unlinked circles. The proportional frequency distribution of pairwise relatedness (Rxy) are shown next to the network for that population. Proportional frequency distribution curves of Rxy for simulated pairs of defined genotypes are shown for comparison.

Rxy frequency distribution curves of simulated pairs show clear differences between the defined relationships, with a dominant density peak at Rxy = 0 in the unrelated pairs, and a progressive right shift of the curve for the simulated half-sib and full-sib pairs (Fig 5). The Rxy frequency distribution curves for the individual UKAGP study populations showed variable right shift of the curve, in association with and blunting or loss of the unrelated peak at Rxy = 0. An equivalent pattern was apparent in the GS50 SW and NE populations (Fig 5). The GS50 populations were analysed separately, as mean Rxy is affected by genetic structure within a sample.

#### Family structure and parentage analysis in COLONY

Fig 5 illustrates networks of individuals linked by first degree relationships (inferred full or half-sibship) in representative UKAGP study populations, based on the best maximum likelihood configurations in COLONY, shown with their respective Rxy frequency distribution curves. Rxy distribution curves for simulated unrelated, half-sib and full-sib pairs are shown for comparison. These networks are characterised by extensive linkage at the inferred half-sib level in all populations. In some study populations, especially those with a right shift of the Rxy frequency distribution curve, networks show dominant inferred half-sibships, some very large, sharing the same inferred parent.

When the GS50 sample was analysed as a single group in COLONY, the patterns of inferred parentage differed between individuals from the SW and NE sampling sites (Fig 4), providing further evidence that the dividing road acts as a barrier to gene flow. The network of COLONY-derived sibships in the GS50 NE and SW samples also showed a loose pedigree linked at the half-sib level, with occasional larger inferred shared-parent sibships, and rare inferred full-sib dyads (Fig 5).

#### Concordance between COLONY and DAPC cluster membership

We found no evidence for intra-population substructure or admixture on STRUCTURE analysis of the individual UKAGP populations (not shown), nor in the GS50 SW or NE samples, consistent with the low F_ST_ results for individual sites. By contrast, DAPC analysis revealed clustering within the all individual study populations, including GS50 (S4 Fig), despite the low F_ST_ results. We hypothesised that patterns of relatedness might underlie this within-population clustering. We therefore compared individual cluster membership in DAPC with inferred sibship and parentage results from COLONY, shown in S5 Fig. The individual membership of large, dominant half-sibships sharing the same inferred parent in COLONY showed striking concordance with that of DAPC clusters. By contrast, for larger populations with looser first-degree relationship networks in COLONY there was poor concordance between COLONY and DAPC cluster membership, illustrated by the results for the large HL population.

## Discussion

The aim of our study was to investigate the genetic status of lowland adders in the UK, in response to concerns about declining numbers, especially affecting small, fragmented populations. We initially adopted a standard panel of microsatellite-based summary statistics, including genetic diversity and the standard F_IS_ measure of inbreeding, to allow comparison with published studies of adders in mainland Europe. We also applied the MTAC criterion of a threshold peak count to categorise study sites into small or large, predicted to be at high or low risk of decline respectively [34]. This initial panel of genetic tests generated a similar pattern of results across all the UKAGP study sites, irrespective of size, although there was a modest decrease in mean allele richness in small populations relative to large. This is likely to have been influenced by the inevitably small sample size, illustrating the difficulty inherent in analysing the unavoidably small sample sizes of the most vulnerable populations.

The interpretation of genetic results requires a biologically relevant comparator, especially for single time-point samples. Estimates of allele richness in the UKAGP study populations were similar to those of the large Belgian population [78], and of populations of lowland adders in Belgium, NE France, and the Netherlands [77], despite the only partially overlapping panels of microsatellite markers used in the different studies. The mtDNA haplotypes of our study populations places them within the Northern phylogenetic clade of European adders [37]. They are therefore likely to have been part of the same post-glacial recolonization process as their counterparts in north-eastern France, in which a central-marginal effect on genetic diversity has been described [77].

Notably, F_IS_ levels were not significantly above zero in any of the UKAGP study populations, irrespective of size, indicating retention of heterozygosity, despite the clear risk of inbreeding depression in fragmented, isolated populations. Our findings are again consistent with published studies of remnant adder populations of varying sizes in mainland Europe [44, 77]. In small isolated populations of species with high philopatry and low vagility, like the adder, mating opportunities will inevitably be biased towards relatives [80, 81], and total avoidance of consanguineous mating could rapidly lead to population extinction [82]. We therefore investigated other measures of consanguinity, a very valuable comparison for the effect of size being provided by the Belgian adder population, isolated but thriving, well-studied, and very much larger than the UKAGP study populations [78].

### Identity by descent: Genetic legacy of population demographic history

In all the UK study populations, genetic estimates of the mean inbreeding coefficient FPED were at a level consistent with the degree of genetic identity expected in the offspring of a one-off mating between half siblings [73]. However, the nature of the defined relationship of simulated pairs of genotypes, whether unrelated, full-sibling or half-sibling, did not appreciably influence FPED estimates. This is in keeping with FPED estimates being a measure of cumulative identity by descent, the embedded genetic legacy of long-standing consanguineous breeding. In the WMids Haplogroup, for example, the shared mtDNA haplotype provides evidence for a historic common ancestor, which could date back to as early as post-glacial colonisation. The Belgian site similarly represents a relict population, probably isolated for more than a century [78].

### Identity by state: Reflection of contemporary relatedness

By contrast pairwise relatedness in simulated pairs of genotypes was significantly and predictably influenced by the defined relationship, providing a very useful template against which to interpret Rxy results for the study populations. We found a range of patterns of relatedness, with variable loss or blunting of the modal unrelated peak seen in the simulated unrelated pairs of genotypes, and right shift of the Rxy distribution curve seen in the half- or full-sibling simulated pairs. Pairwise Rxy thus generated the most informative results in our study, with the potential caveat that results may be influenced by cryptic genetic differentiation within the sample. Repeat studies will be necessary to determine trends and timescales in changing patterns of relatedness.

COLONY results, like Rxy, provide a snapshot estimate of contemporary relatedness between individuals within the sample, but in the format of best maximum likelihood combinations of inferred genotypes of sibling and parent-offspring dyads. This approach generated dramatic networks of inferred half-sibships in our study populations. However, in the absence of pedigree data to inform COLONY analysis, loose networks of inferred half sibships may simply reflect identity by state, rather than true first-degree relationships, especially for inferred half sibships in larger populations [76]. While we sought to minimise this phenomenon by applying stringent parameters for sibship assignment in COLONY, it is likely to have been exacerbated by the presence of a high level of background relatedness, as well as by the relatively limited number of genetic markers. This high level of relatedness is also the likely explanation for the unexpectedly low estimates of Ne by the sibship method for the GS50 populations.

We found the membership of inferred dominant large sibships in small populations to be concordant with the assignment of individuals to clusters in DAPC, in keeping with DAPC clusters reflecting allele frequency patterns driven by a polygynandrous mating system in a small population, rather than discrete panmictic subpopulations. An equivalent phenomenon of clustering in DAPC, but not STRUCTURE, has also been described in the Prairie rattlesnake *(Crotalus viridis)* [83]. This is an important consideration when DAPC is used to investigate cryptic genetic structure within consanguineous populations.

### Inbreeding depression: Protective effect of polyandry

As a single year snapshot, our study is only a starting point, and inevitably limited by population size, and thus sample size, especially in the study of the most vulnerable populations. We have nevertheless exposed a previously undocumented degree of consanguinity in wild adder populations, despite their showing no increase in homozygosity to suggest inbreeding depression. In models of inbreeding, a system of half-sib mating is more likely to maintain heterozygosity than one of maximum avoidance of inbreeding [84, 85]. Polyandry, which is widespread in taxa of live-bearing snakes [86], including the adder [87–89], may thus represent a protective mechanism against inbreeding [82, 90]. Interestingly, Mourier et al (2013) described a very similar network pattern of extensive pairwise relatedness between individuals of the sicklefin lemon shark (*Negaprion acutidens*) in French Polynesia [91]. Despite the biological differences, there are clear similarities in the reproductive ecology between the taxa, the lemon shark also being viviparous, of low female fecundity, with a polyandrous mating system and limited distribution [91].

### Inbreeding depression: Size matters

While polyandry may delay, it will not prevent the eventual loss of heterozygosity in isolated populations, where movement of adders is prevented by loss of connectivity between patches of fragmented habitat. It is therefore interesting to compare the large, thriving Belgian site, with high levels of relatedness but no loss of heterozygosity, with the well-documented Swedish population of adders with unequivocal evidence for inbreeding depression [14–16]. Both sites have been isolated for more than a century by agricultural landscape, are situated some 20km from neighbouring adder populations [15, 78], and both would fulfil the MTAC definition of a large population [34]. However, at 1570ha, the Belgian site is significantly larger than its 20ha Swedish counterpart, which provides the likely explanation for the difference in inbreeding. As the Belgian site has more recently been subjected to asymmetric fragmentation by the truncating road, it will be especially interesting to monitor the genetic status of the smaller NE fragment in comparison with its larger SW counterpart unless gene flow can be restored across the truncating road.

### Safety in numbers: Demographic vulnerability of smaller adder populations

“Unfortunately, the best way to find tipping points so far has been to cross them–a dangerous proposition” [92]. Our findings suggest that genetic factors are unlikely to be the direct cause of the observed decline in small populations of adders. Instead, small populations may already be “doomed to extinction by demographic factors before genetic effects act strongly” [93], representing the “living dead” [94, 95], where continuation of a population or metapopulation becomes demographically impossible. For example, the reproductive ecology of adders renders small populations profoundly vulnerable to stochastic sex bias [96–98]. In adders, males are the actively mate-seeking sex [99, 100], with only a short interval of female sexual receptiveness, and thus limited time available for mating, while female adders have low lifetime fecundity, with high fitness costs of reproduction [101, 9, 10], suggesting particular vulnerability of small adder populations to a limiting number of females. Conversely, a relative reduction in males would be predicted to impact on any protective effect of polyandry against inbreeding. Sampling over a limited period precluded an accurate field assessment of sex ratios in our study, but we are addressing this question of breeding sex ratios in ongoing work.

### Future studies

We are using radio-telemetry of adult snakes to inform habitat management, especially with respect to connectivity [100], in combination with ongoing genetic monitoring of study populations. This will allow us to investigate the reproductive success of potential mating connections, generating very interesting data with respect to the breeding system in this secretive species, including assortative mate choice, overt or cryptic. Pedigree information will also enhance the interpretation of results in COLONY, including estimates of the effective numbers of breeding adults.

We plan to use genomic sequencing to increase the number of informative markers available, especially important for genetic diversity and pedigree studies. In addition, the development of a genomic SNP panel will help to increase consistency and comparability across sites and laboratories, currently hampered by the limited numbers of microsatellite markers, and potential inconsistencies in their application [102–104]. This will be especially important in decision making and post-release monitoring in any future adder translocations, whether for reasons of mitigation or conservation [105–107]. A panel of genomic SNPs will also allow the investigation of heterozygosity affecting different loci [104], facilitating the study of inbreeding at the whole genome level. In addition, the pattern of runs of homozygosity [108–112] will help to elucidate the demographic history of this fascinating species, as well as the identification of potential targets of selection [113].

## Conclusions

Our results suggest that the most immediate threat to small adder populations is demographic rather than genetic. For larger populations high levels of relatedness indicate that genetic factors are likely to represent a real threat, albeit less imminent, but also less visible and thus more insidious [22]. Continuing monitoring will be essential to determine the urgency and nature of intervention. Our study thus underscores the need for a systematic, evidence-driven approach in conservation planning for adder populations, whether healthy or declining, integrating population genetics and traditional ecology [33]. In this the “true cost of loss and degradation of habitat” [13] should not be neglected, including public engagement to reduce persecution by changing the public perception of snakes [114]. Attempts at genetic or demographic rescue may be similarly doomed unless such underlying factors can be addressed [115].

## Supporting information

S1 Fig Phylogenetic tree of UKAGP showing mtDNA haplotypes relative to European clades. Bootstrap consensus tree of UKAGP concatenated Cytb/CR haplotypes (500 replicates) relative to homologous sequences in Genbank.
(PPTX)

S2 Fig Effect of *locprior* on STRUCTURE results. Bar charts showing proportional membership coefficients of individuals to each of the inferred clusters for K = 3–6, grouped according to their study population, with locprior option (left) and without (right). Hierarchical clustering is apparent with both approaches. The colour schemes differ between bar charts.
(PPTX)

S3 Fig Concordance between STRUCTURE and DAPC. Pie charts for each population of the WMids Haplogroup, showing the proportion of group membership assigned probabilistically to K = 3 or K = 6 clusters in analysis applying the *locprior* option. In each panel the populations are superimposed on a Venn diagram of overlapping circles according to their broad proportional membership of the three clusters inferred in STRUCTURE for K = 3. The colour schemes are independent for STRUCTURE and DAPC. Top left panel: pie charts for K = 3 in STRUCTURE. Top right panel: pie charts for K = 3 in DAPC. Bottom left panel: pie charts for K = 6 in STRUCTURE. Top right panel: pie charts for K = 6 in DAPC.
(PPTX)

S4 Fig DAPC clustering in individual populations. DAPC scatterplots (K = 3) for individual study populations with sample size ≥ 10, including GS50 SW and NE, showing clearly separated clusters.
(PPTX)

S5 Fig Concordance between COLONY and DAPC. For each population, the network of COLONY-inferred sibship dyads is shown, together with a table (upper) of inferred parentage for each individual, and their assignment to one of the clusters of individuals linked at a minimum of half-sibling level. Dominant hypothetical parents are highlighted in the parentage table and network. The lower table for each population shows bar plots of the probability of assignment of each individual (in same order as in COLONY) to DAPC clusters. For population CH (top left), the assignment of individuals to K = 2 clusters in DAPC is concordant with the COLONY-defined clusters, assignment of individuals to the largest of K = 3 clusters in DAPC is concordant with the hypothetical dominant parent of the inferred family structure in COLONY. Different patterns of concordance are evident in populations MHS, BM, EH and MF. By contrast, for the larger population HL (bottom right), the membership of DAPC clusters shows poor concordance with the inferred parentage of the larger, looser COLONY network.
(PPTX)

S1 Table Genbank sequences used in alignment for phylogenetic tree. (PPTX)

S2 Table Effect on summary statistics of removal of loci with missing data. (DOCX)

S3 Table Matrix of mean pairwise relatedness within and between populations. (DOCX)

S4 Table Excel spreadsheet of UKAGP microsatellite genotypes. (XLSX)

S1 Text Alignment of UKAGP concatenated Cytb/CR haplotypes (fas format). (TXT)
