# Supplementary material for: Genetic and demographic vulnerability of adder populations: Results of a genetic study in mainland Britain
Source: PLoS One. 2020 Apr 20;15(4):e0231809. doi: 10.1371/journal.pone.0231809 (PMC7170227; doi:10.1371/journal.pone.0231809)
Supplement: S1 Fig — Bootstrap consensus tree of UKAGP concatenated Cytb/CR haplotypes (500 replicates) relative to homologous sequences in Genbank. (PPTX) [file pone.0231809.s001.pptx]

## Slide 1
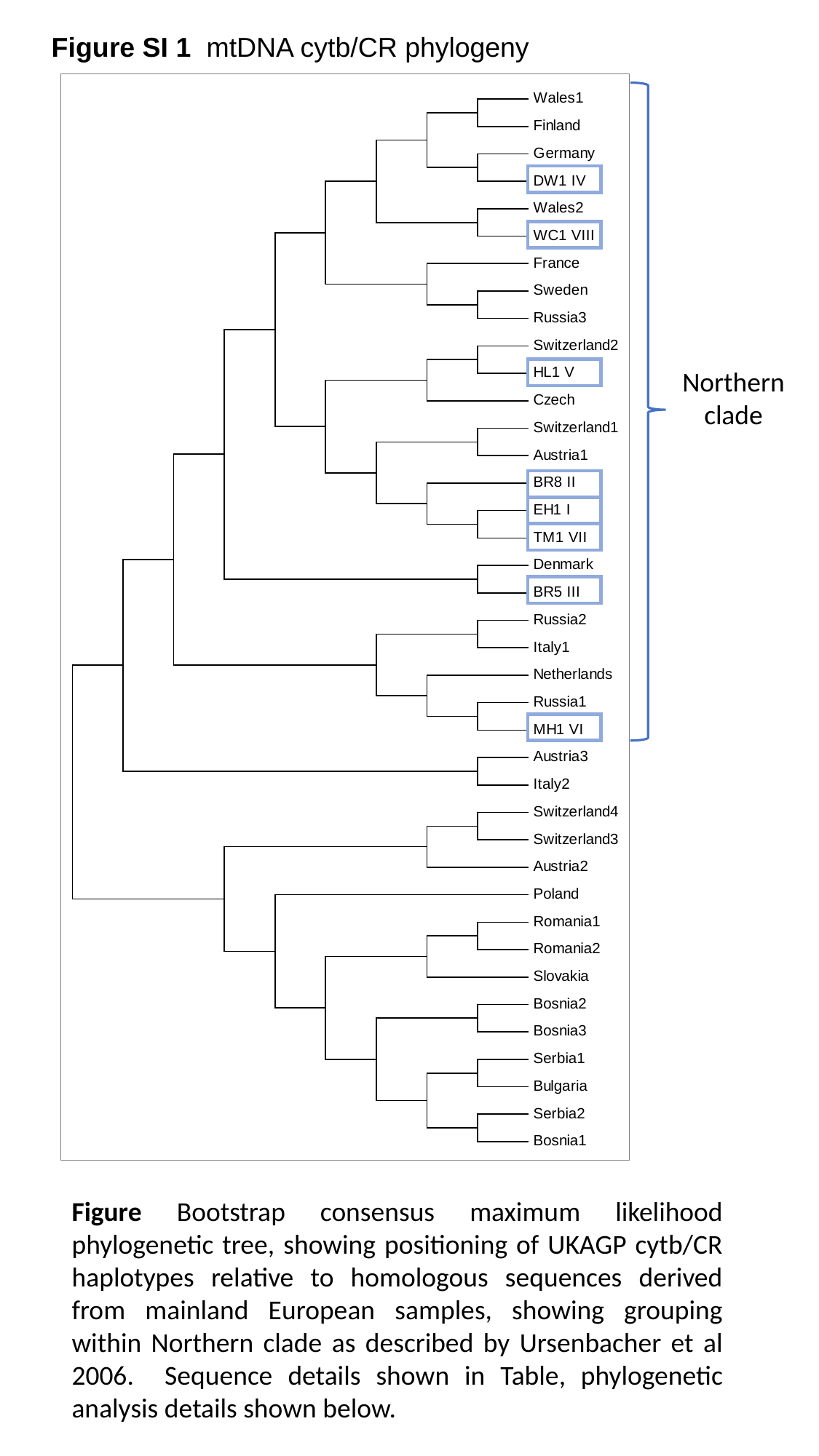

Figure SI 1 mtDNA cytb/CR phylogeny
Northern clade
Figure Bootstrap consensus maximum likelihood phylogenetic tree, showing positioning of UKAGP cytb/CR haplotypes relative to homologous sequences derived from mainland European samples, showing grouping within Northern clade as described by Ursenbacher et al 2006. Sequence details shown in Table, phylogenetic analysis details shown below.

## Slide 2
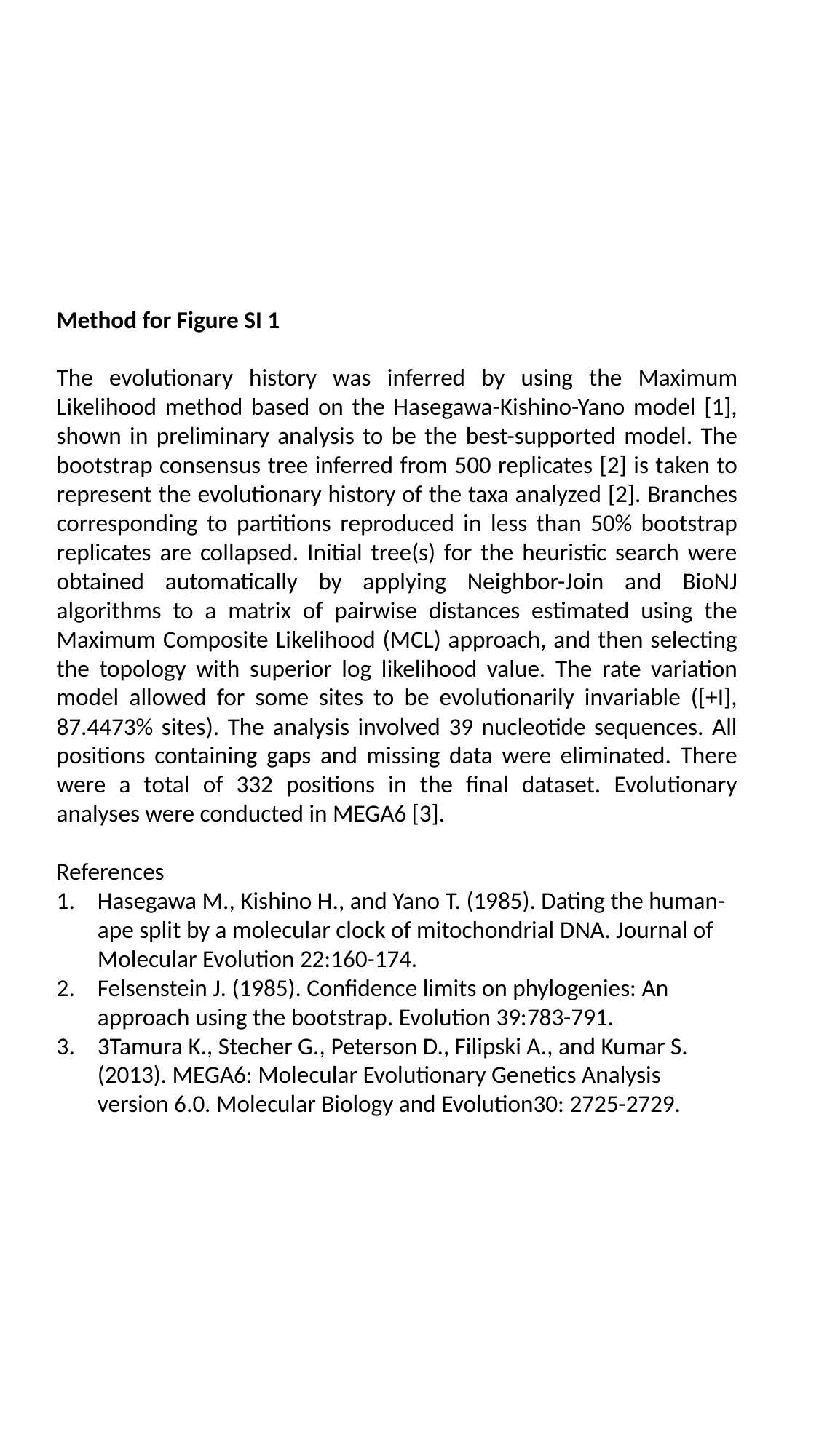

Method for Figure SI 1
The evolutionary history was inferred by using the Maximum Likelihood method based on the Hasegawa-Kishino-Yano model [1], shown in preliminary analysis to be the best-supported model. The bootstrap consensus tree inferred from 500 replicates [2] is taken to represent the evolutionary history of the taxa analyzed [2]. Branches corresponding to partitions reproduced in less than 50% bootstrap replicates are collapsed. Initial tree(s) for the heuristic search were obtained automatically by applying Neighbor-Join and BioNJ algorithms to a matrix of pairwise distances estimated using the Maximum Composite Likelihood (MCL) approach, and then selecting the topology with superior log likelihood value. The rate variation model allowed for some sites to be evolutionarily invariable ([+I], 87.4473% sites). The analysis involved 39 nucleotide sequences. All positions containing gaps and missing data were eliminated. There were a total of 332 positions in the final dataset. Evolutionary analyses were conducted in MEGA6 [3].
References
Hasegawa M., Kishino H., and Yano T. (1985). Dating the human-ape split by a molecular clock of mitochondrial DNA. Journal of Molecular Evolution 22:160-174.
Felsenstein J. (1985). Confidence limits on phylogenies: An approach using the bootstrap. Evolution 39:783-791.
3Tamura K., Stecher G., Peterson D., Filipski A., and Kumar S. (2013). MEGA6: Molecular Evolutionary Genetics Analysis version 6.0. Molecular Biology and Evolution30: 2725-2729.
