# Supplementary material for: Genetic and demographic vulnerability of adder populations: Results of a genetic study in mainland Britain
Source: PLoS One. 2020 Apr 20;15(4):e0231809. doi: 10.1371/journal.pone.0231809 (PMC7170227; doi:10.1371/journal.pone.0231809)
Supplement: S2 Fig — Bar charts showing proportional membership coefficients of individuals to each of the inferred clusters for K = 3–6, grouped according to their study population, with locprior option (left) and without (right). Hierarchical clustering is apparent with both approaches. The colour schemes differ between bar charts. (PPTX) [file pone.0231809.s002.pptx]

## Slide 1
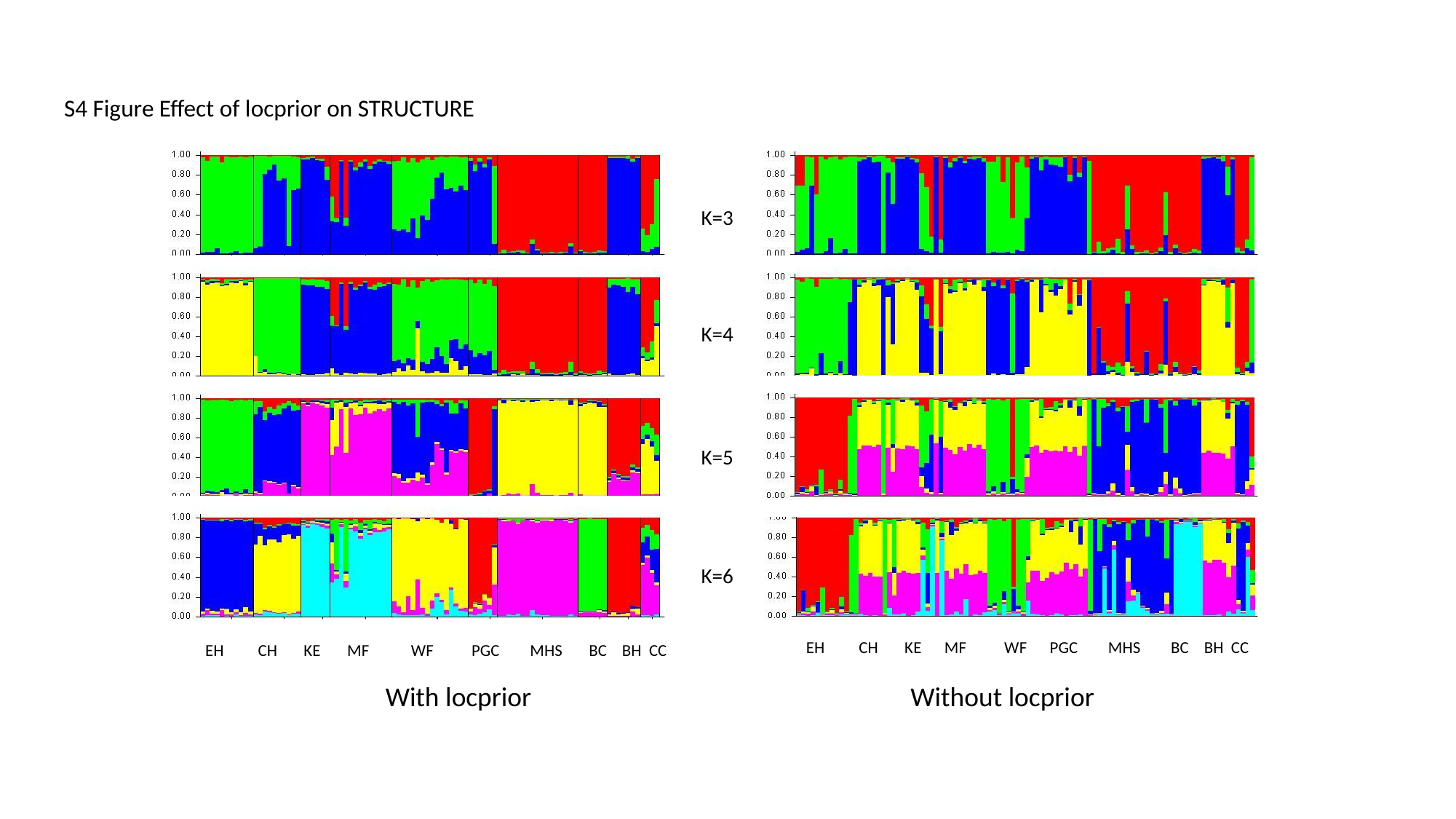

S4 Figure Effect of locprior on STRUCTURE
K=3
K=4
K=5
K=6
EH CH KE MF WF PGC MHS BC BH CC
EH CH KE MF WF PGC MHS BC BH CC
With locprior
Without locprior
