# Supplementary material for: Genetic and demographic vulnerability of adder populations: Results of a genetic study in mainland Britain
Source: PLoS One. 2020 Apr 20;15(4):e0231809. doi: 10.1371/journal.pone.0231809 (PMC7170227; doi:10.1371/journal.pone.0231809)
Supplement: S3 Fig — Pie charts for each population of the WMids Haplogroup, showing the proportion of group membership assigned probabilistically to K = 3 or K = 6 clusters in analysis applying the locprior option. In each panel the populations are superimposed on a Venn diagram of overlapping circles according to their broad proportional membership of the three clusters inferred in STRUCTURE for K = 3. The colour schemes are independent for STRUCTURE and DAPC. Top left panel: pie charts for K = 3 in STRUCTURE. Top right panel: pie charts for K = 3 in DAPC. Bottom left panel: pie charts for K = 6 in STRUCTURE. Top right panel: pie charts for K = 6 in DAPC. (PPTX) [file pone.0231809.s003.pptx]

## Slide 1
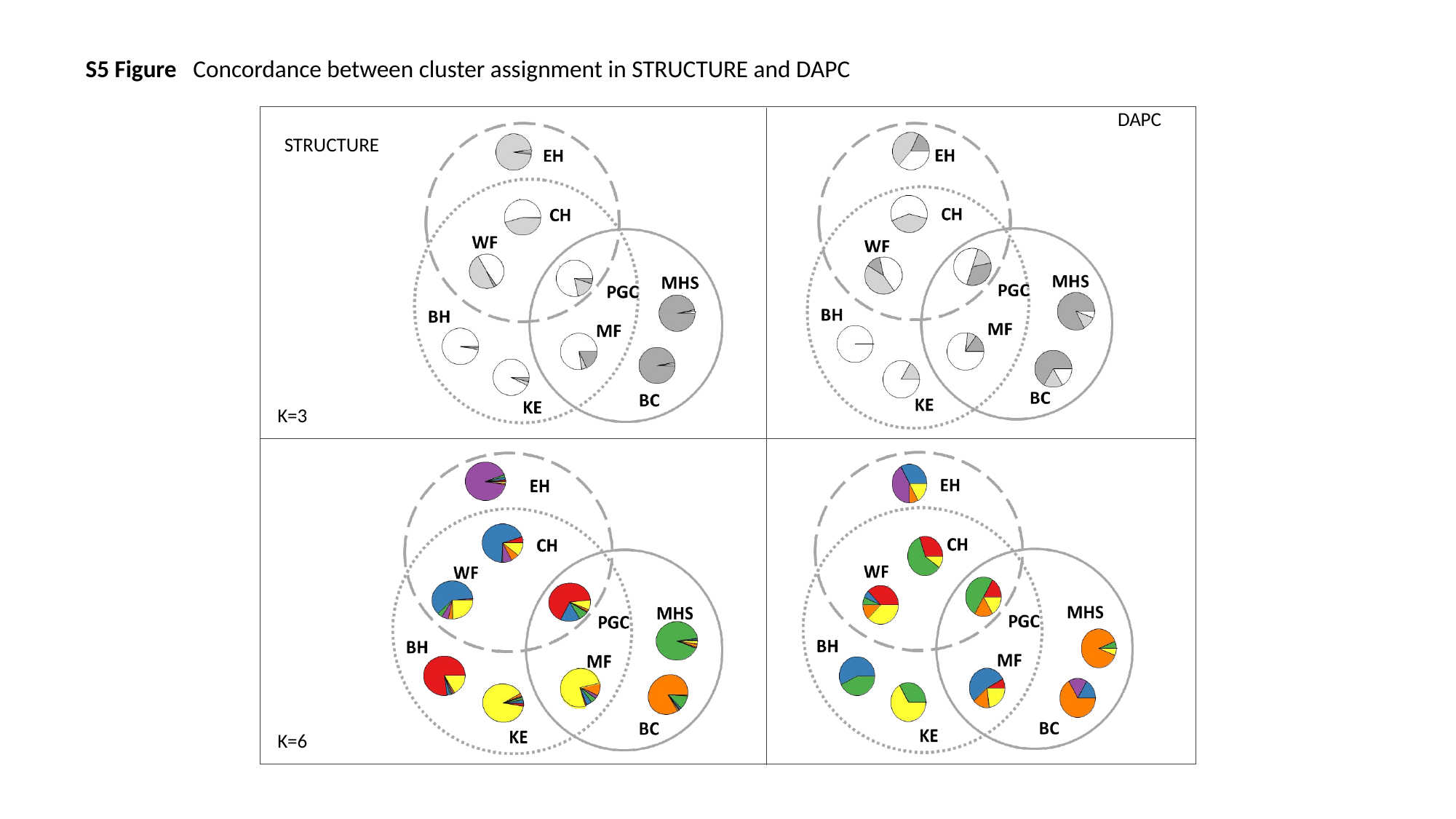

S5 Figure Concordance between cluster assignment in STRUCTURE and DAPC
DAPC
STRUCTURE
K=3
K=6
