# Supplementary figures and images for: Genetic and demographic vulnerability of adder populations: Results of a genetic study in mainland Britain
Source: PLoS One. 2020 Apr 20;15(4):e0231809. doi: 10.1371/journal.pone.0231809 (PMC7170227; doi:10.1371/journal.pone.0231809)

## Slide 1
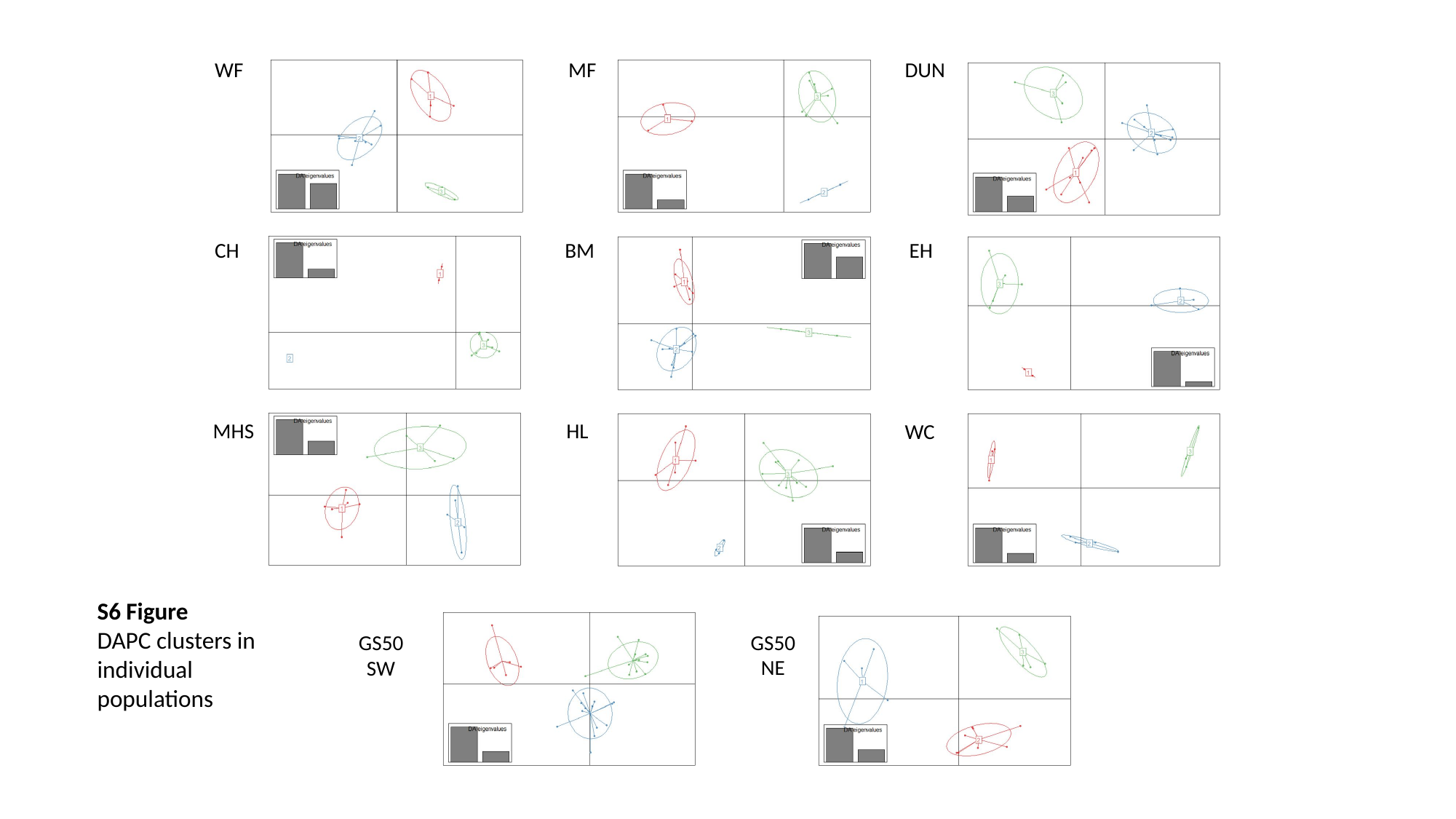

WF
MF
DUN
EH
CH
BM
MHS
HL
WC
S6 Figure
DAPC clusters in individual populations
GS50 NE
GS50 SW

Supplement: S4 Fig — DAPC scatterplots (K = 3) for individual study populations with sample size ≥ 10, including GS50 SW and NE, showing clearly separated clusters. (PPTX) [file pone.0231809.s004.pptx]
