# Supplementary material for: Genetic and demographic vulnerability of adder populations: Results of a genetic study in mainland Britain
Source: PLoS One. 2020 Apr 20;15(4):e0231809. doi: 10.1371/journal.pone.0231809 (PMC7170227; doi:10.1371/journal.pone.0231809)
Supplement: S5 Fig — For each population, the network of COLONY-inferred sibship dyads is shown, together with a table (upper) of inferred parentage for each individual, and their assignment to one of the clusters of individuals linked at a minimum of half-sibling level. Dominant hypothetical parents are highlighted in the parentage table and network. The lower table for each population shows bar plots of the probability of assignment of each individual (in same order as in COLONY) to DAPC clusters. For population CH (top left), the assignment of individuals to K = 2 clusters in DAPC is concordant with the COLONY-defined clusters, assignment of individuals to the largest of K = 3 clusters in DAPC is concordant with the hypothetical dominant parent of the inferred family structure in COLONY. Different patterns of concordance are evident in populations MHS, BM, EH and MF. By contrast, for the larger population HL (bottom right), the membership of DAPC clusters shows poor concordance with the inferred parentage of the larger, looser COLONY network. (PPTX) [file pone.0231809.s005.pptx]

## Slide 1
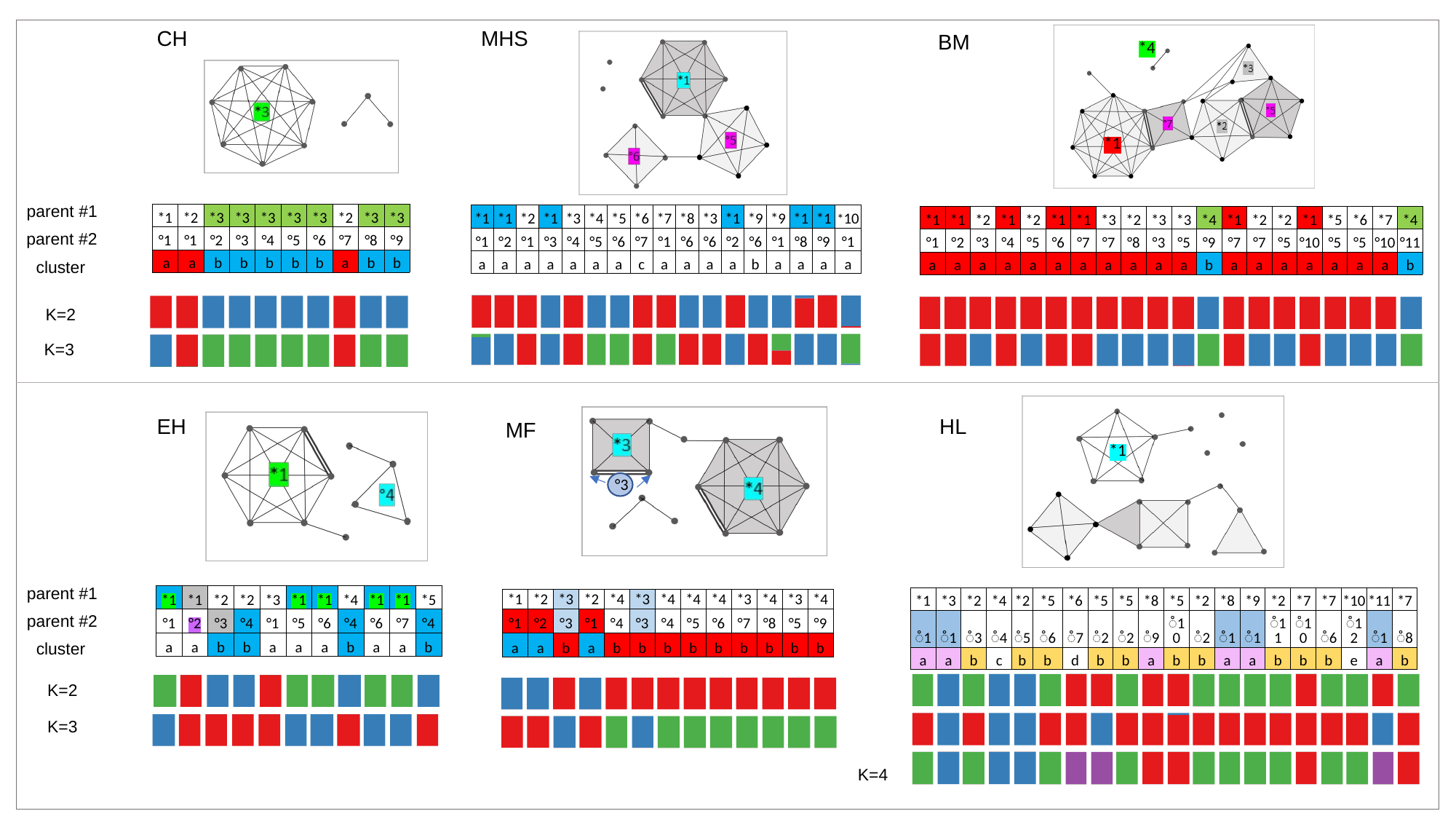

CH
MHS
BM
*4
*1
parent #1
parent #2
 cluster
| \*1 | \*2 | \*3 | \*3 | \*3 | \*3 | \*3 | \*2 | \*3 | \*3 |
| --- | --- | --- | --- | --- | --- | --- | --- | --- | --- |
| °1 | °1 | °2 | °3 | °4 | °5 | °6 | °7 | °8 | °9 |
| a | a | b | b | b | b | b | a | b | b |
| \*1 | \*1 | \*2 | \*1 | \*3 | \*4 | \*5 | \*6 | \*7 | \*8 | \*3 | \*1 | \*9 | \*9 | \*1 | \*1 | \*10 |
| --- | --- | --- | --- | --- | --- | --- | --- | --- | --- | --- | --- | --- | --- | --- | --- | --- |
| °1 | °2 | °1 | °3 | °4 | °5 | °6 | °7 | °1 | °6 | °6 | °2 | °6 | °1 | °8 | °9 | °1 |
| a | a | a | a | a | a | a | c | a | a | a | a | b | a | a | a | a |
| \*1 | \*1 | \*2 | \*1 | \*2 | \*1 | \*1 | \*3 | \*2 | \*3 | \*3 | \*4 | \*1 | \*2 | \*2 | \*1 | \*5 | \*6 | \*7 | \*4 |
| --- | --- | --- | --- | --- | --- | --- | --- | --- | --- | --- | --- | --- | --- | --- | --- | --- | --- | --- | --- |
| °1 | °2 | °3 | °4 | °5 | °6 | °7 | °7 | °8 | °3 | °5 | °9 | °7 | °7 | °5 | °10 | °5 | °5 | °10 | °11 |
| a | a | a | a | a | a | a | a | a | a | a | b | a | a | a | a | a | a | a | b |
K=2
K=3
°3
EH
HL
MF
*1
parent #1
parent #2
 cluster
| \*1 | \*1 | \*2 | \*2 | \*3 | \*1 | \*1 | \*4 | \*1 | \*1 | \*5 |
| --- | --- | --- | --- | --- | --- | --- | --- | --- | --- | --- |
| °1 | °2 | °3 | °4 | °1 | °5 | °6 | °4 | °6 | °7 | °4 |
| a | a | b | b | a | a | a | b | a | a | b |
| \*1 | \*3 | \*2 | \*4 | \*2 | \*5 | \*6 | \*5 | \*5 | \*8 | \*5 | \*2 | \*8 | \*9 | \*2 | \*7 | \*7 | \*10 | \*11 | \*7 |
| --- | --- | --- | --- | --- | --- | --- | --- | --- | --- | --- | --- | --- | --- | --- | --- | --- | --- | --- | --- |
| ̊1 | ̊1 | ̊3 | ̊4 | ̊5 | ̊6 | ̊7 | ̊2 | ̊2 | ̊9 | ̊10 | ̊2 | ̊1 | ̊1 | ̊11 | ̊10 | ̊6 | ̊12 | ̊1 | ̊8 |
| a | a | b | c | b | b | d | b | b | a | b | b | a | a | b | b | b | e | a | b |
| \*1 | \*2 | \*3 | \*2 | \*4 | \*3 | \*4 | \*4 | \*4 | \*3 | \*4 | \*3 | \*4 |
| --- | --- | --- | --- | --- | --- | --- | --- | --- | --- | --- | --- | --- |
| °1 | °2 | °3 | °1 | °4 | °3 | °4 | °5 | °6 | °7 | °8 | °5 | °9 |
| a | a | b | a | b | b | b | b | b | b | b | b | b |
K=2
K=3
K=4
