# Supplementary material for: Genetic and demographic vulnerability of adder populations: Results of a genetic study in mainland Britain
Source: PLoS One. 2020 Apr 20;15(4):e0231809. doi: 10.1371/journal.pone.0231809 (PMC7170227; doi:10.1371/journal.pone.0231809)
Supplement: S1 Table — (PPTX) [file pone.0231809.s006.pptx]

## Slide 1
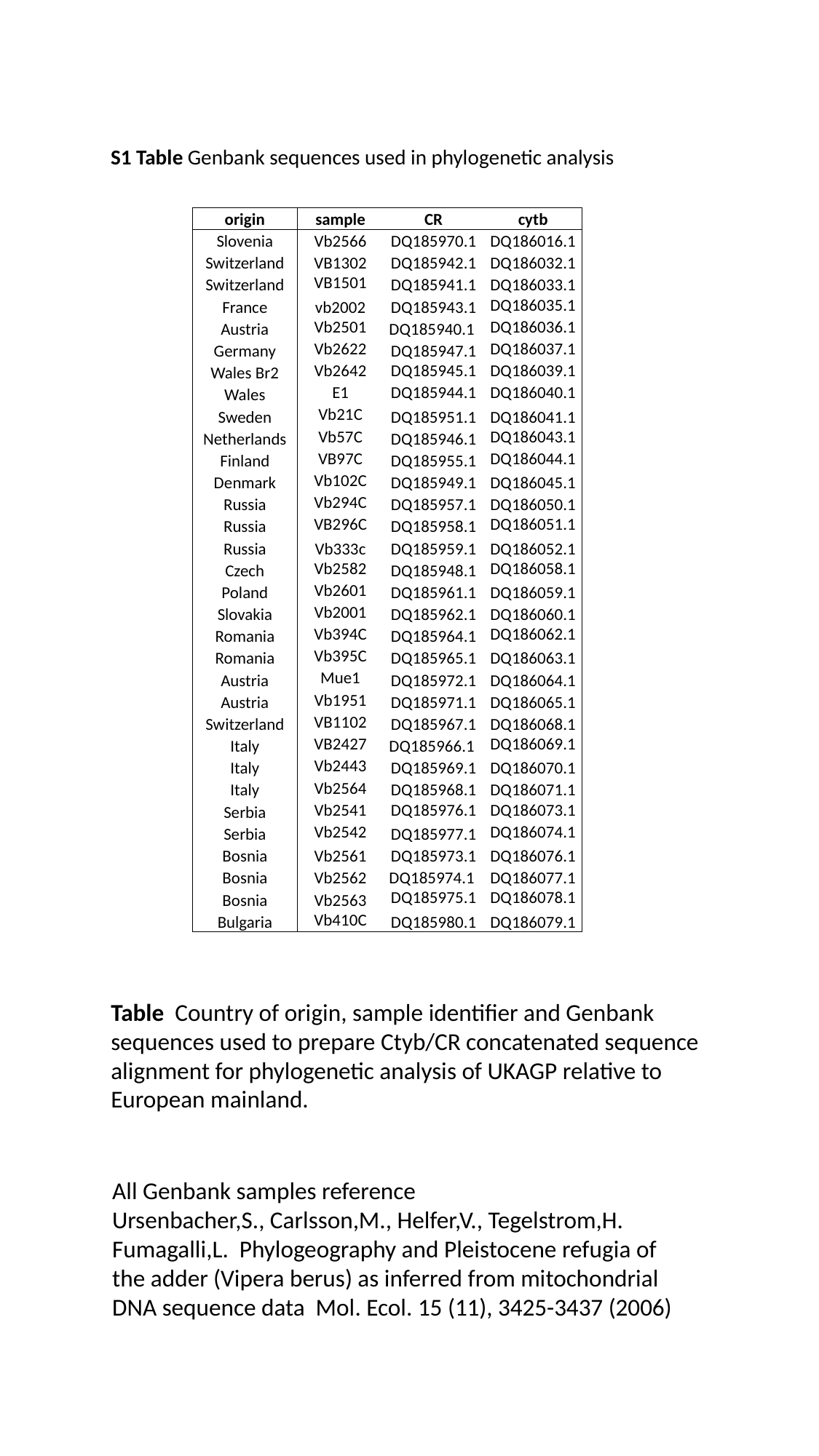

S1 Table Genbank sequences used in phylogenetic analysis
| origin | sample | CR | cytb |
| --- | --- | --- | --- |
| Slovenia | Vb2566 | DQ185970.1 | DQ186016.1 |
| Switzerland | VB1302 | DQ185942.1 | DQ186032.1 |
| Switzerland | VB1501 | DQ185941.1 | DQ186033.1 |
| France | vb2002 | DQ185943.1 | DQ186035.1 |
| Austria | Vb2501 | DQ185940.1 | DQ186036.1 |
| Germany | Vb2622 | DQ185947.1 | DQ186037.1 |
| Wales Br2 | Vb2642 | DQ185945.1 | DQ186039.1 |
| Wales | E1 | DQ185944.1 | DQ186040.1 |
| Sweden | Vb21C | DQ185951.1 | DQ186041.1 |
| Netherlands | Vb57C | DQ185946.1 | DQ186043.1 |
| Finland | VB97C | DQ185955.1 | DQ186044.1 |
| Denmark | Vb102C | DQ185949.1 | DQ186045.1 |
| Russia | Vb294C | DQ185957.1 | DQ186050.1 |
| Russia | VB296C | DQ185958.1 | DQ186051.1 |
| Russia | Vb333c | DQ185959.1 | DQ186052.1 |
| Czech | Vb2582 | DQ185948.1 | DQ186058.1 |
| Poland | Vb2601 | DQ185961.1 | DQ186059.1 |
| Slovakia | Vb2001 | DQ185962.1 | DQ186060.1 |
| Romania | Vb394C | DQ185964.1 | DQ186062.1 |
| Romania | Vb395C | DQ185965.1 | DQ186063.1 |
| Austria | Mue1 | DQ185972.1 | DQ186064.1 |
| Austria | Vb1951 | DQ185971.1 | DQ186065.1 |
| Switzerland | VB1102 | DQ185967.1 | DQ186068.1 |
| Italy | VB2427 | DQ185966.1 | DQ186069.1 |
| Italy | Vb2443 | DQ185969.1 | DQ186070.1 |
| Italy | Vb2564 | DQ185968.1 | DQ186071.1 |
| Serbia | Vb2541 | DQ185976.1 | DQ186073.1 |
| Serbia | Vb2542 | DQ185977.1 | DQ186074.1 |
| Bosnia | Vb2561 | DQ185973.1 | DQ186076.1 |
| Bosnia | Vb2562 | DQ185974.1 | DQ186077.1 |
| Bosnia | Vb2563 | DQ185975.1 | DQ186078.1 |
| Bulgaria | Vb410C | DQ185980.1 | DQ186079.1 |
Table Country of origin, sample identifier and Genbank sequences used to prepare Ctyb/CR concatenated sequence alignment for phylogenetic analysis of UKAGP relative to European mainland.
All Genbank samples reference
Ursenbacher,S., Carlsson,M., Helfer,V., Tegelstrom,H. Fumagalli,L. Phylogeography and Pleistocene refugia of the adder (Vipera berus) as inferred from mitochondrial DNA sequence data Mol. Ecol. 15 (11), 3425-3437 (2006)
