# Supplementary material for: Genetic and demographic vulnerability of adder populations: Results of a genetic study in mainland Britain
Source: PLoS One. 2020 Apr 20;15(4):e0231809. doi: 10.1371/journal.pone.0231809 (PMC7170227; doi:10.1371/journal.pone.0231809)
Supplement: S2 Table — (DOCX) [file pone.0231809.s007.docx]

| **site** | **Ar** | | | **Ho** | | | **Hs** | | | **Fis** | | |
| --- | --- | --- | --- | --- | --- | --- | --- | --- | --- | --- | --- | --- |
|  | **8 loci** | **7 i** | **7 ii** | **8 loci** | **7 i** | **7 ii** | **8 loci** | **7 i** | **7 ii** | **8 loci** | **7 i** | **7 ii** |
| **EH** | 2.424 | 2.366 | 2.452 | 0.631 | 0.604 | 0.604 | 0.642 | 0.624 | 0.653 | 0.017 | 0.031 | 0.074 |
| **CH** | 2.751 | 2.695 | 2.734 | 0.699 | 0.713 | 0.656 | 0.734 | 0.715 | 0.734 | 0.047 | 0.003 | 0.106 |
| **WC** | 2.792 | 2.805 | 2.743 | 0.792 | 0.788 | 0.762 | 0.732 | 0.733 | 0.72 | -0.081 | -0.075 | -0.057 |
| **KE** | 2.671 | 2.695 | 2.767 | 0.673 | 0.662 | 0.698 | 0.755 | 0.756 | 0.797 | 0.109 | 0.124 | 0.125 |
| **MF** | 2.731 | 2.76 | 2.835 | 0.664 | 0.641 | 0.675 | 0.732 | 0.74 | 0.772 | 0.093 | 0.133 | 0.125 |
| **BM** | 2.608 | 2.59 | 2.688 | 0.711 | 0.653 | 0.717 | 0.691 | 0.684 | 0.713 | -0.029 | 0.045 | -0.006 |
| **WF** | 2.747 | 2.761 | 2.764 | 0.748 | 0.736 | 0.73 | 0.732 | 0.734 | 0.736 | -0.023 | -0.003 | 0.007 |
| **MH** | 2.597 | 2.568 | 2.676 | 0.608 | 0.593 | 0.6 | 0.694 | 0.679 | 0.726 | 0.124 | 0.127 | 0.174 |
| **PGC** | 2.613 | 2.543 | 2.557 | 0.646 | 0.595 | 0.595 | 0.72 | 0.708 | 0.716 | 0.104 | 0.16 | 0.169 |
| **DUN** | 2.602 | 2.605 | 2.63 | 0.704 | 0.671 | 0.677 | 0.691 | 0.691 | 0.699 | -0.018 | 0.028 | 0.031 |
| **HL** | 2.717 | 2.745 | 2.741 | 0.732 | 0.694 | 0.703 | 0.717 | 0.726 | 0.72 | -0.021 | 0.045 | 0.024 |
| **MHS** | 2.595 | 2.573 | 2.601 | 0.62 | 0.617 | 0.583 | 0.689 | 0.679 | 0.689 | 0.099 | 0.092 | 0.154 |
| **BC** | 2.433 | 2.395 | 2.524 | 0.646 | 0.619 | 0.667 | 0.67 | 0.659 | 0.706 | 0.036 | 0.06 | 0.056 |
| **BH** | 2.613 | 2.658 | 2.658 | 0.767 | 0.733 | 0.762 | 0.676 | 0.69 | 0.688 | -0.133 | -0.063 | -0.107 |
| **TM** |  |  | 1.986 |  |  | 0.457 |  |  | 0.5 |  |  | 0.086 |
| **CC** |  | 2.357 |  |  | 0.679 |  |  | 0.589 |  |  | -0.152 |  |
|  |  |  |  |  |  |  |  |  |  |  |  |  |
|  |  |  |  |  |  |  |  |  |  |  |  |  |

**S3 Table Effect of removal of CA71 or Vu4 results on summary statistics for UKAGP study populations**

7i: no CA71; 7ii: no Vu4

Ar; allele richness; H0 : observed heterozygosity; Hs : gene density (observed heterozygosity)
