# Supplementary material for: Genetic and demographic vulnerability of adder populations: Results of a genetic study in mainland Britain
Source: PLoS One. 2020 Apr 20;15(4):e0231809. doi: 10.1371/journal.pone.0231809 (PMC7170227; doi:10.1371/journal.pone.0231809)
Supplement: S3 Table — (DOCX) [file pone.0231809.s008.docx]

|  | **EH** | **CH** | **WC** | **KE** | **MF** | **BM** | **WF** | **MH** | **PGC** | **DUN** | **HL** | **MHS** | **BC** | **BH** | **TM** | **CC** |
| --- | --- | --- | --- | --- | --- | --- | --- | --- | --- | --- | --- | --- | --- | --- | --- | --- |
| **EH** | 0.268 |  |  |  |  |  |  |  |  |  |  |  |  |  |  |  |
| **CH** | 0.06 | 0.192 |  |  |  |  |  |  |  |  |  |  |  |  |  |  |
| **WC** | 0.043 | 0.128 | 0.14 |  |  |  |  |  |  |  |  |  |  |  |  |  |
| **KE** | 0.034 | 0.074 | 0.114 | 0.221 |  |  |  |  |  |  |  |  |  |  |  |  |
| **MF** | 0.071 | 0.063 | 0.05 | 0.127 | 0.165 |  |  |  |  |  |  |  |  |  |  |  |
| **BM** | 0.066 | 0.08 | 0.116 | 0.131 | 0.096 | 0.232 |  |  |  |  |  |  |  |  |  |  |
| **WF** | 0.072 | 0.102 | 0.054 | 0.076 | 0.08 | 0.063 | 0.135 |  |  |  |  |  |  |  |  |  |
| **MH** | 0.038 | 0.059 | 0.064 | 0.027 | 0.063 | 0.051 | 0.064 | 0.171 |  |  |  |  |  |  |  |  |
| **PGC** | 0.044 | 0.075 | 0.102 | 0.037 | 0.057 | 0.096 | 0.074 | 0.046 | 0.186 |  |  |  |  |  |  |  |
| **DUN** | 0.022 | 0.058 | 0.044 | 0.057 | 0.052 | 0.055 | 0.055 | 0.05 | 0.036 | 0.202 |  |  |  |  |  |  |
| **HL** | 0.048 | 0.061 | 0.063 | 0.07 | 0.051 | 0.065 | 0.047 | 0.042 | 0.039 | 0.11 | 0.177 |  |  |  |  |  |
| **MHS** | 0.075 | 0.046 | 0.03 | 0.038 | 0.065 | 0.04 | 0.081 | 0.068 | 0.081 | 0.025 | 0.036 | 0.235 |  |  |  |  |
| **BC** | 0.04 | 0.044 | 0.018 | 0.025 | 0.061 | 0.035 | 0.035 | 0.049 | 0.008 | 0.05 | 0.04 | 0.106 | 0.268 |  |  |  |
| **BH** | 0.029 | 0.052 | 0.057 | 0.069 | 0.097 | 0.056 | 0.051 | 0.041 | 0.09 | 0.066 | 0.065 | 0.023 | 0.052 | 0.25 |  |  |
| **TM** | 0.043 | 0.063 | 0.039 | 0.027 | 0.032 | 0.037 | 0.075 | 0.04 | 0.157 | 0.044 | 0.043 | 0.026 | 0.003 | 0.127 | 0.377 |  |
| **CC** | 0.102 | 0.085 | 0.057 | 0.006 | 0.02 | 0.078 | 0.034 | 0.043 | 0.071 | 0.019 | 0.016 | 0.116 | 0.115 | 0.044 | 0.02 | 0.31 |

**S7 Table**  **Matrix of mean pairwise relatedness within and between populations**

Shaded boxes denote mean intra-population Rxy, unshaded mean Rxy between populations. All populations showed significant within-population relatedness
